# Experimental and Numerical Evaluation of Recycled Thermoplastic Composites for Structural Applications in Sustainable Construction

**DOI:** 10.3390/ma19204331

**Published:** 2026-10-15

**Authors:** Karla Parada-Rocha, Andrés Guzmán, Carlos Parada-Rocha, Nemesio Daza-Marquez

**Affiliations:** 1 Department of Civil Engineering, Universidad Simón Bolívar, Barranquilla 080001, Colombia; karla.parada@unisimon.edu.co; 2 Department of Civil and Environmental Engineering, Universidad del Norte, Barranquilla 081007, Colombia; cparadaa@uninorte.edu.co (C.P.-R.); nemesiod@uninorte.edu.co (N.D.-M.)

**Keywords:** agro-industrial waste, mechanical characterization, low-rise housing, load-bearing members, finite element modeling, carbon footprint, polymer blending, inverse identification

## Abstract

**Highlights:**

**What are the main findings?**

- Agro-industrial residues supplied 31% of the recycled thermoplastic feedstock experimentally characterized.
- Inverse fitting gave effective fractions of 26% high-density and 36% low-density polyethylene, and 38% polypropylene.
- Numerical optimization with acrylonitrile butadiene styrene increased the predicted tensile modulus by 91.7%.

**What are the implications of the main findings?**

- Estimated material-related emissions are about 59% lower under the adopted inventory assumptions.
- Single-story housing is a potential application, subject to experimental validation of members and connections.

**Abstract:**

This study presents an experimental and numerical framework for evaluating recycled thermoplastic composites for structural applications in sustainable construction. The base material (BM), comprising recycled high-density polyethylene (HDPE), low-density polyethylene (LDPE), and polypropylene (PP), was characterized in tension and compression; inverse mechanical identification yielded assigned domain fractions of 26%, 36%, and 38%, respectively. Numerical incorporation of acrylonitrile butadiene styrene (ABS) resulted in an optimized composite (CMo: 13% HDPE, 18% LDPE, 19% PP, and 50% ABS), increasing the predicted tensile elastic modulus from 871.36 MPa to 1670.4 MPa (91.7%). Thermal modeling illustrated the BM temperature distribution under prescribed boundary temperatures, while a preliminary structural assessment considered CMo beams, columns, and roof-support members for single-story housing and identified insufficient lateral stiffness under seismic loading. An inventory-based comparison yielded approximately 59% lower estimated CO_2_-equivalent emissions for the CMo structural system than for the reinforced-concrete and steel reference, subject to the adopted emission factors and differing source boundaries. The framework supports formulation selection for further investigation, although experimental validation of the CMo, members, and connections remains necessary before structural suitability can be established.

## 1. Introduction

Concrete remains the most widely used construction material; however, its environmental impact is strongly influenced by cement production, together with the extraction, processing, and transportation of raw materials. The cement and concrete industries are responsible for a significant share of global anthropogenic CO_2_ emissions, with cement production alone contributing approximately 8% of the global total [1]. This environmental burden has intensified the search for alternative materials and construction systems with lower embodied carbon.

In parallel, plastic waste represents another major environmental challenge. Global plastic production reached approximately 460 million tonnes in 2019, while plastic waste generation amounted to 353 million tonnes; only about 9% of this waste was ultimately recycled [2]. Mechanical recycling and upcycling therefore offer opportunities to transform these waste streams into higher-value engineering products. Recycled thermoplastics such as polypropylene (PP), high-density polyethylene (HDPE), and low-density polyethylene (LDPE) are particularly attractive because of their low density, corrosion resistance, processability, and recyclability. Nevertheless, their relatively low stiffness, sensitivity to temperature, time-dependent behavior, and variability resulting from repeated processing continue to restrict their use in load-bearing structural applications [3,4,5].

Recent research has demonstrated that the mechanical performance of recycled thermoplastics can be improved through polymer blending, reinforcement, controlled manufacturing, and numerical optimization. Recycled PP/HDPE blends have been experimentally evaluated for civil engineering applications, demonstrating that their mechanical response strongly depends on the proportions of the constituent polymers [6]. Similarly, recycled HDPE reinforced with milled glass-fiber-reinforced polymer has shown substantial improvements in stiffness [7]. More recently, the upcycling of glass-fiber-reinforced polypropylene waste through injection molding has demonstrated that appropriate processing can increase tensile and flexural performance while reducing internal defects [8]. These studies illustrate the potential of recycled thermoplastics as engineering materials while also showing that composition, manufacturing conditions, reinforcement, and interfacial compatibility play critical roles in their resulting properties.

Most applications of recycled polymers in construction, however, have focused on their incorporation into concrete, nonstructural products, panels, blocks, or hybrid systems rather than on their direct use as primary load-bearing members. Earlier thermoplastic construction systems proposed interconnected panels and structural components, but their load-bearing capacity commonly depended on concrete infill, metallic components, fiber reinforcement, or other stiffening mechanisms [9,10]. Recycled plastic beams and other digitally manufactured components have also been proposed, although additional materials or internal reinforcement has generally been required to provide sufficient structural stiffness [11]. Therefore, an important challenge remains: not simply to reuse plastic waste, but to develop recycled polymer-based materials capable of providing sufficient mechanical performance for structural elements while reducing dependence on conventional concrete and steel systems.

The performance of polymer blends is also affected by compatibility between the constituent phases. Previous research has shown that incompatibility between polymers may cause heterogeneous phase distributions, poor interfacial adhesion, and reductions in mechanical performance. Compatibilizers and fiber reinforcement can mitigate these effects and substantially improve tensile and flexural properties. Processing and recycling routes can likewise modify surface characteristics and the interaction between recycled constituents and polymer matrices [12]. Recent finite element studies of recycled thermoplastic blends, including HDPE/PP systems, further demonstrate the usefulness of numerical approaches for predicting their mechanical response and assessing their potential as matrices for structural composite applications [13]. In the present investigation, fibers and compatibilizers were intentionally not incorporated, allowing the contribution of the recycled thermoplastic blend and the subsequent incorporation of acrylonitrile butadiene styrene (ABS) to be evaluated independently. Fiber reinforcement and compatibilization are therefore regarded as subsequent stages in the development of the proposed material.

Despite these advances, limited information is available on the use of heterogeneous recycled PP, HDPE, and LDPE waste streams as the primary constituents of load-bearing structural materials, particularly when their original proportions are unknown at the collection stage. Furthermore, few studies integrate experimental characterization, inverse mechanical identification of the effective polymer composition, finite element modeling, material optimization, structural-system analysis, and environmental assessment within a single methodology. This gap is particularly relevant for thermoplastic waste originating from agro-industrial activities, where substantial waste streams may be generated but their potential for higher-value structural reuse remains insufficiently explored.

Accordingly, the novelty of this study lies not merely in the reuse of recycled plastics, but in the integrated experimental and numerical development of a recycled thermoplastic composite intended for structural applications. The base material (BM) consists of HDPE and LDPE originating partly from agro-industrial residues generated in banana-producing regions of northern Colombia, together with PP from urban waste streams. Because the initial proportions of these constituents were not available from the recycling process, their effective composition was identified by matching the experimentally measured tensile response with finite element simulations. The mechanically identified BM was subsequently modified through the incorporation of ABS to obtain an optimized composite material (CMo). The working hypothesis is that controlled ABS incorporation can substantially increase the stiffness of the recycled thermoplastic blend while preserving the low density and environmental advantages associated with the use of recycled polymers.

To test this hypothesis, the study experimentally characterizes the tensile and compressive behavior of the BM and uses finite element modeling to reproduce its mechanical response and identify its effective HDPE/LDPE/PP composition. ABS is subsequently incorporated numerically to evaluate its influence on the mechanical and thermal response of the resulting CMo. The optimized material is then implemented in a preliminary structural assessment of beams, columns, and roof-support elements for a low-rise residential system, followed by an estimation of the associated CO_2_ emissions relative to an equivalent reinforced concrete solution. The structural analysis presented herein constitutes a preliminary feasibility assessment rather than a complete qualification of the material for structural design; experimental flexural characterization, full-scale testing, connection performance, durability, and comprehensive seismic validation remain subjects for future investigation.

## 2. Materials and Methods

**Figure 1:**
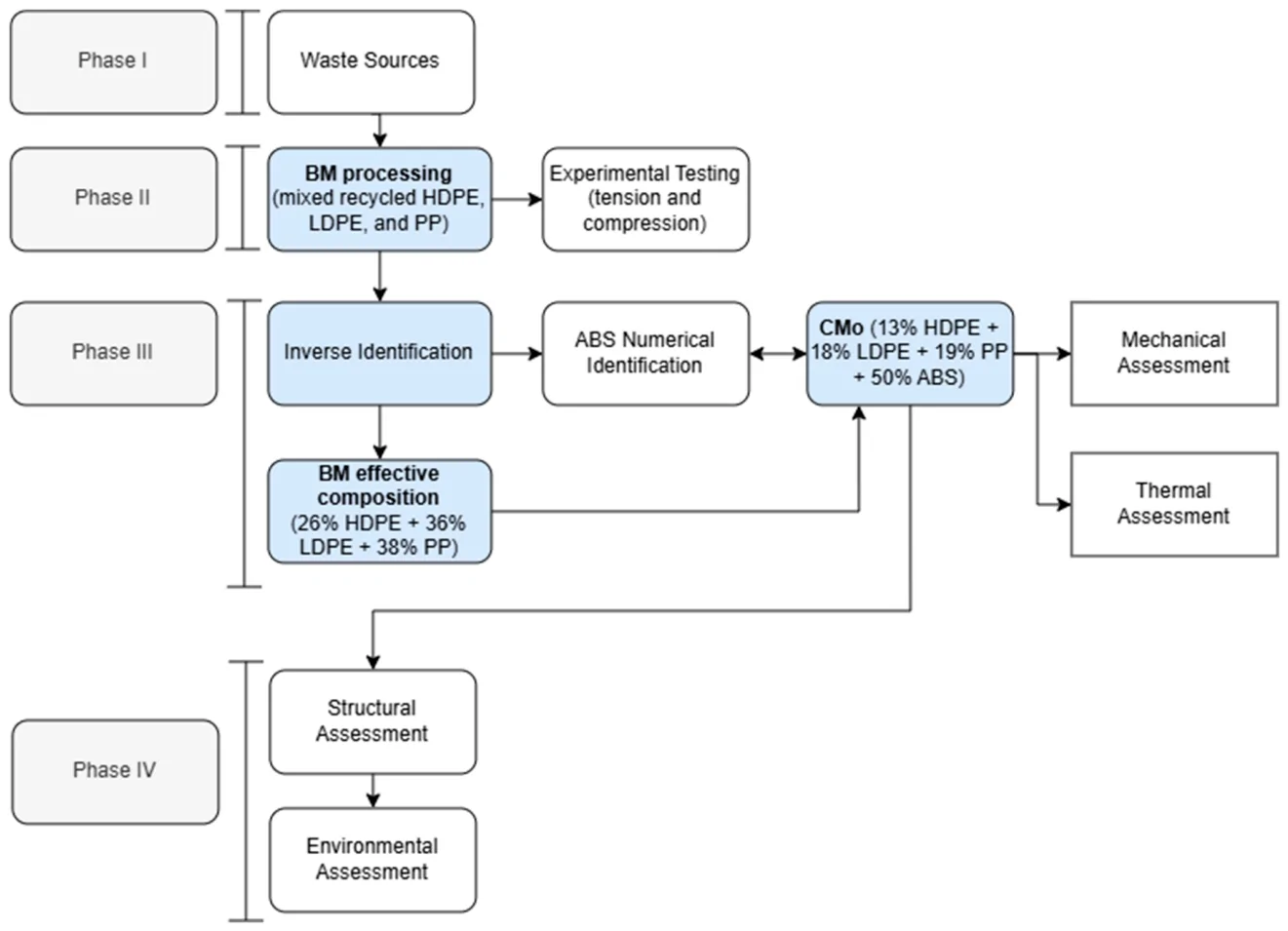
Methodological framework of the study, including material sourcing and processing, experimental characterization of the BM, inverse identification of its effective composition, numerical optimization with ABS to obtain the CMo, and the subsequent structural and environmental assessments.

The methodological framework of this study is organized into four sequential phases, as summarized in Figure 1. Phase I comprises the sourcing and preparation of the recycled thermoplastic base material (BM). Phase II includes experimental tensile and compressive characterization of the BM. In Phase III, finite element models are calibrated against the experimental response to identify the effective mechanical composition of the BM and subsequently evaluate the incorporation of acrylonitrile butadiene styrene (ABS) to obtain an optimized composite material (CMo), including its mechanical and thermal response. Finally, Phase IV uses the numerically optimized CMo properties in a preliminary structural assessment of a low-rise residential system and an environmental comparison with an equivalent reinforced concrete solution. This sequence distinguishes the experimentally characterized material from the numerically optimized composite and defines the scope of each stage of the investigation.

### 2.1. Phase I: Material Sourcing and Preparation

HDPE and LDPE were sourced from industrial waste generated in the banana-producing regions of the Magdalena and La Guajira departments (Colombia), while PP was obtained from urban solid waste in the Magdalena department. Approximately 31% of the thermoplastics considered in this study originated from agro-industrial residues, whereas the remaining fraction came from urban waste streams. These three polymers constituted the base material (BM) experimentally characterized in this research. Acrylonitrile butadiene styrene (ABS) was not part of the original BM supply; instead, it was introduced later during the numerical optimization stage to define the optimized composite material (CMo).

**Figure 2:**
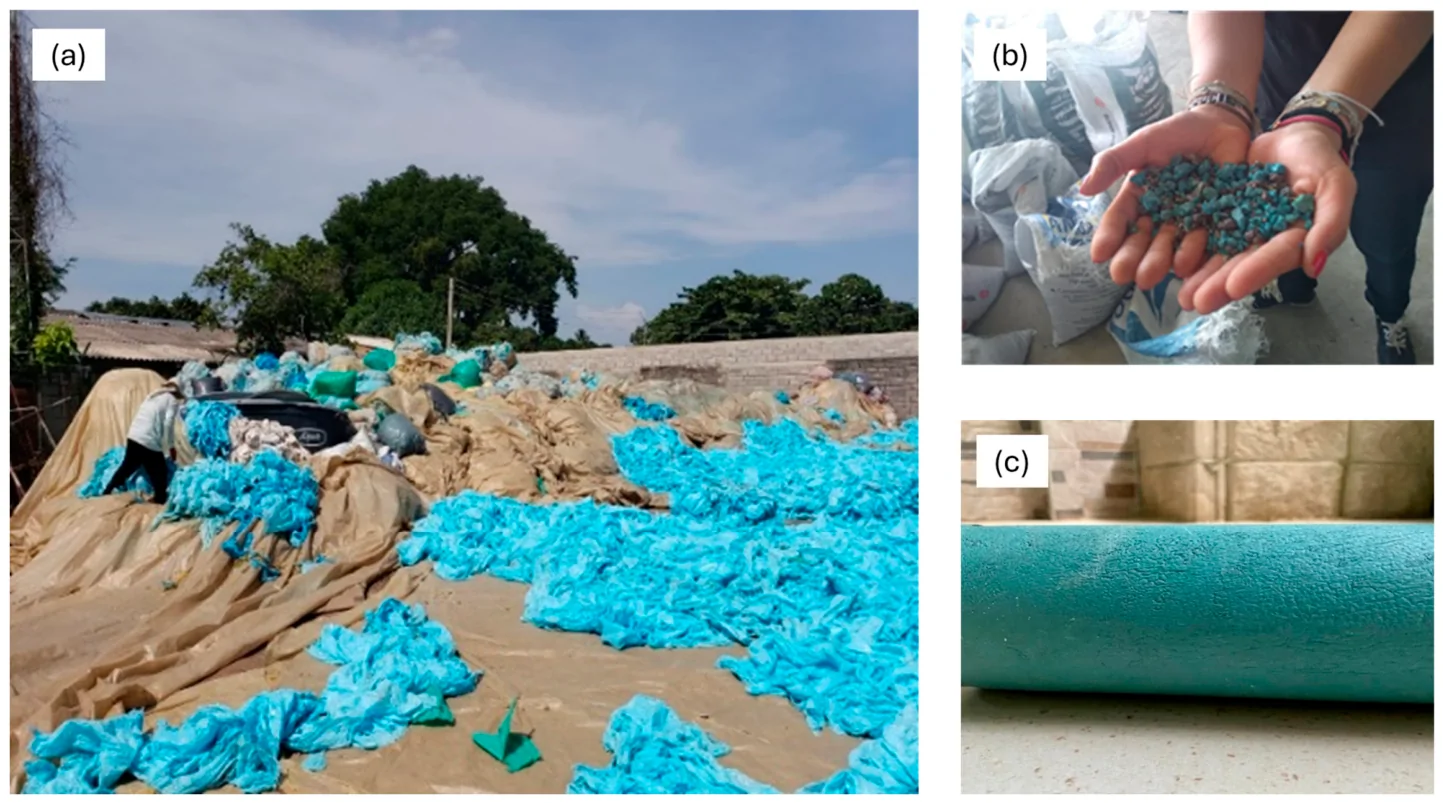
Sourcing and industrial preparation of the base material (BM): (**a**) recycled thermoplastic collection, (**b**) crushed thermoplastic feedstock, and (**c**) molded BM supplied for experimental characterization.

The recycled thermoplastics supplied for the BM were sorted, washed, and sun-dried on an impermeable surface to minimize the presence of soil and organic contaminants (Figure 2a). They were then industrially crushed to obtain an amorphous particulate material (Figure 2b), which was subsequently melted and molded into solid sections with the required shapes (Figure 2c). It should be noted that Figure 2a–c illustrate only the industrial preparation of the base material (BM: HDPE, LDPE, and PP); ABS was incorporated later only during the numerical optimization stage used to define the CMo. The exact industrial processing parameters, including temperature, mixing conditions, pressure, and cooling rate, were not provided by the manufacturer and therefore could not be independently controlled in this study.

At the time of collection, the exact weight fractions of HDPE, LDPE and PP in the BM were unknown. Direct compositional characterization by techniques such as differential scanning calorimetry (DSC), Fourier-transform infrared spectroscopy (FTIR), or thermogravimetric analysis (TGA) was not possible within the scope of this study. Therefore, the polymer proportions were estimated through inverse mechanical identification: the HDPE/LDPE/PP fractions in the numerical models were systematically varied until the simulated elastic tensile response matched the experimentally measured elastic modulus of the BM. The resulting proportions—26% HDPE, 36% LDPE, and 38% PP– were adopted as an effective mechanically identified composition for subsequent numerical analyses and should not be interpreted as chemically measured constituent fractions.

**Figure 3:**
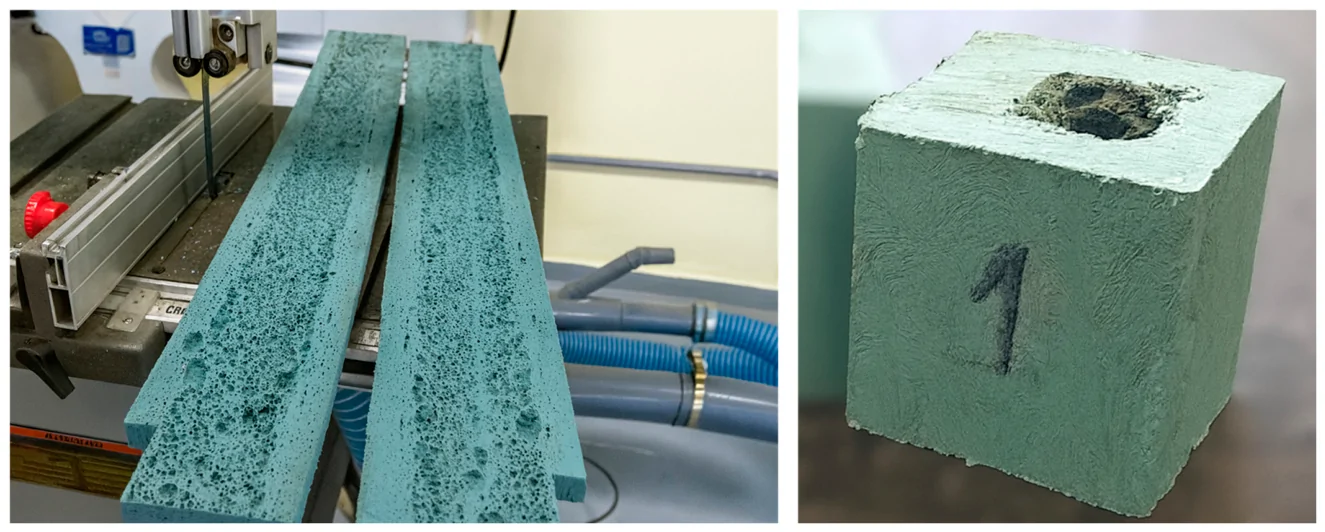
Internal porosity observed in the base material (BM) during specimen cutting.

The BM was supplied by the collaborating company as rectangular and square solid sections, which were subsequently cut to prepare specimens for tensile and compression testing. During cutting, noticeable internal porosity was observed predominantly in the central region of some sections, as shown in Figure 3. Because the industrial processing parameters were not available to the research team, the specific origin of this porosity could not be established. The observed voids were therefore documented as an inherent feature of the supplied recycled material that may contribute to variability in its measured mechanical response.

### 2.2. Phase II: Experimental Characterization

Phase II comprised the laboratory characterization of the base material (BM) under uniaxial tension and compression. Five tensile specimens and three cubic compression specimens were prepared from the molded BM supplied by the collaborating company. Tensile testing was conducted according to ASTM D638-14 [14], while compression testing followed the procedure described in ASTM C109/C109M [15], as detailed in Section 2.2.1 and Section 2.2.2. Only the BM was experimentally tested in this phase; the optimized composite material (CMo) was subsequently evaluated numerically in Phase III.

#### 2.2.1. Tensile Testing

Five BM specimens were prepared for tensile testing in accordance with ASTM D638-14 [14]. The specimen geometry followed the standard requirements, while the actual dimensions of the reduced section were measured individually before testing. For the four specimens retained for quantitative analysis, the average thickness ranged from 5.92 to 6.41 mm and the average width from 18.93 to 19.54 mm, with an initial gauge length of 57 mm. One of the five specimens was excluded from the quantitative analysis because fracture occurred outside the reduced section and coincided with the presence of visible organic material in the specimen. Accordingly, the tensile results reported in this study correspond to four valid specimens (n = 4).

**Figure 4:**
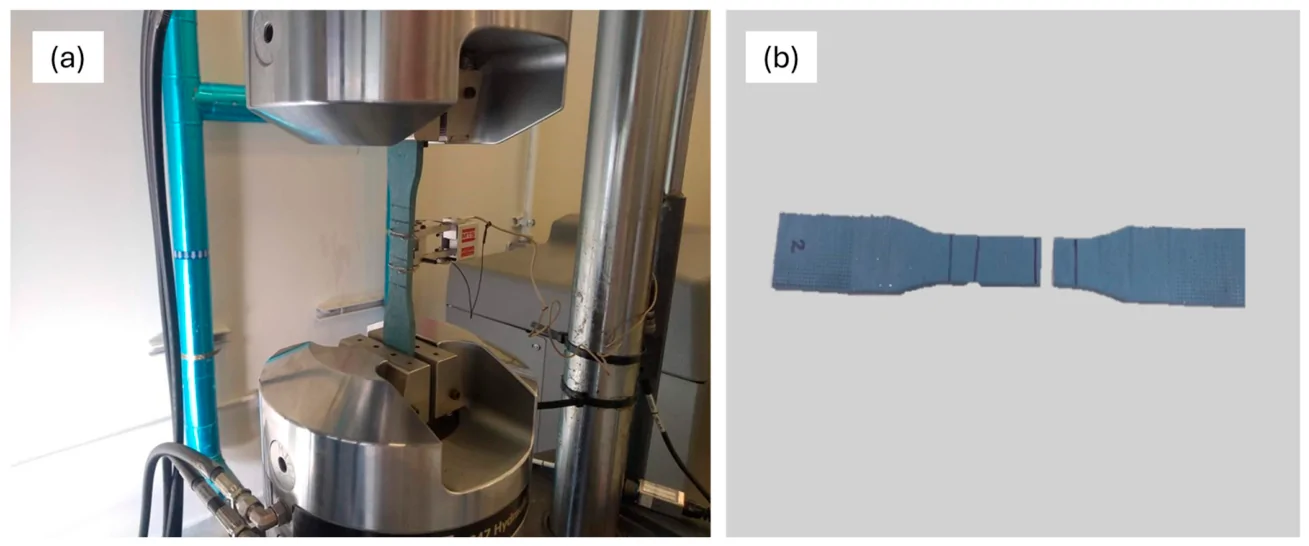
Tensile testing of the BM specimens: (**a**) specimen positioned in the testing machine before loading and (**b**) representative specimen after testing.

Tensile tests were performed using an Instron 8802 servo-hydraulic testing system (Instron^®^, Norwood, MA, USA), equipped with an axial extensometer from the MTS 634.11F series (MTS Systems Corporation, Eden Prairie, MN, USA) (Figure 4a). The Instron 8802 system has a maximum dynamic force capacity of ±250 kN. The tensile tests were conducted under displacement control. During testing, axial force, extensometer strain, and axial displacement were recorded. The axial extensometer, with a nominal gauge length of 25 mm, provided the strain measurements used to construct the stress–strain response and determine the elastic modulus of the BM. Initial and final specimen dimensions were additionally measured to determine elongation and reduction in cross-sectional area, with a representative specimen after testing shown in Figure 4b.

For each valid specimen, engineering stress (*σ*) was calculated from the measured axial force and the initial cross-sectional area, while engineering strain (*ε*) was obtained directly from the axial extensometer signal. The resulting stress–strain data were used to characterize the tensile response of the BM and to determine its elastic modulus from the initial quasi-linear region. The initial cross-sectional area, percentage elongation after fracture, and percentage reduction in area were calculated using Equations (1)–(3), respectively.

(1) $$A_{0}=\bar{t}\bar{w}$$

(2) $$\mathrm{EL}=\frac{l_{f}-l_{0}}{l_{0}}\times 100$$

(3) $$\mathrm{AR}=\frac{A_{0}-A_{f}}{A_{0}}\times 100$$

where *A*_0_ is the initial cross-sectional area [mm^2^]; $\bar{t}$ is the average specimen thickness [mm]; $\bar{w}$ is the average specimen width [mm]; *l*_0_ and *l_f_* are the initial and final gauge lengths [mm], respectively; *A_f_* is the final cross-sectional area measured at the fracture region [mm^2^]; *EL* is the elongation after fracture [%]; and *AR* is the reduction in cross-sectional area [%].

The mean tensile stress–strain curve reported in Section 3.1.1 was obtained by averaging the stress and strain values of the four valid specimens at corresponding data-acquisition points; it therefore represents the mean response of the tested group and not the record of an individual specimen.

#### 2.2.2. Compression Testing

Compression testing was performed on three BM specimens (n = 3) using an adapted procedure based on ASTM C109 [15]. Although this standard was developed for determining the compressive strength of hydraulic-cement mortars, its 50 mm cubic specimen configuration was adopted as a methodological reference to characterize the compressive response of the recycled thermoplastic material. Three nominally 50 × 50 × 50 mm cubic specimens were cut from a molded BM section using a band saw. The bulk density of the BM was determined experimentally by measuring the mass and external dimensions of the specimens and calculating the corresponding mass-to-volume ratio. A bulk density of 970 kg/m^3^ was obtained and adopted for the subsequent comparisons. During testing, the specimens did not reach physical failure but exhibited substantial recoverable deformation. Therefore, the tests were terminated at applied loads of approximately 76.6 kN to avoid specimen instability, misalignment, or potential damage to the testing equipment.

Compression tests were performed using a Shimadzu universal testing machine (Shimadzu Corporation, Kyoto, Japan) with a nominal maximum compression capacity of 500 kN. Load application, control, and data acquisition were managed through an Automax control console (Controls^®^ Group, Milan, Italy), with a force scale division of 0.01 kN. During each test, the applied compressive load was continuously recorded and subsequently divided by the measured loaded cross-sectional area of each specimen to obtain the corresponding compressive stress (*σ*).

### 2.3. Phase III: Numerical Modeling and Material Optimization

Phase III comprised six finite element models developed in COMSOL Multiphysics^®^ 5.2a [16] to characterize the numerical response of the BM and subsequently define the optimized composite material (CMo). The mechanical models were implemented using the Solid Mechanics interface, whereas the thermal analysis used the Heat Transfer in Solids interface. Models 1 and 2 addressed the linear tensile response of the BM through inverse identification and specimen-scale verification, respectively; Model 3 evaluated its nonlinear tensile response. Model 4 assessed the numerical incorporation of ABS into the mechanically identified BM; Model 5 evaluated the thermal response of the BM and CMo under an imposed temperature gradient; and Model 6 investigated their compressive behavior. Experimental data obtained in Phase II provided the reference response for the numerical characterization of the BM prior to material optimization. Accordingly, the properties predicted for the CMo in this phase represent numerical estimates and were not experimentally validated in the present study.

#### 2.3.1. General FEM Formulation and Numerical Settings

For the linear-range mechanical simulations, the polymer constituents were represented as isotropic linear-elastic materials within the Solid Mechanics interface. Their constitutive response was defined by Young’s modulus E and Poisson’s ratio ν, according to the isotropic elasticity relation (4).

(4) $$\sigma =\frac{\mathrm{E\nu}}{\left(1+\nu \right)\left(1-2\nu \right)}\mathrm{tr}\left(\varepsilon \right)I+\frac{E}{1+\nu }\varepsilon$$

where *σ* is the Cauchy stress tensor, *ε* is the strain tensor, *I* is the identity tensor, and tr(*ε*) denotes the trace of the strain tensor. The values of *E* and *ν* assigned to each constituent or effective material are specified for the corresponding models below. The loading procedure and response assessment for Model 3 are described separately in Section 2.3.4.

The numerical models were discretized using the physics-controlled meshing approach available in COMSOL Multiphysics^®^, with the global element-size setting defined as Normal [16]. The automatic meshing procedure accounts for the active physics interfaces and the geometric characteristics of the modeled domains, introducing local variations in element size where required by geometric features. No user-defined local refinement regions or manually prescribed element-size transitions were introduced. Because the geometries considered in this study were relatively simple, the numerical analyses relied on this automatically generated discretization, and a separate mesh-convergence study was not performed.

Unless otherwise specified for an individual model, the default discretization settings of the corresponding COMSOL physics interfaces were retained. For Solid Mechanics, the displacement field was represented using the default quadratic serendipity formulation, whereas the Heat Transfer in Solids interface employed quadratic Lagrange shape functions for the temperature field [16]. Numerical quadrature and the associated integration points were automatically selected by COMSOL according to the element and discretization orders. These software-controlled finite element settings were not manually modified in the present models.

#### 2.3.2. Model 1: Inverse Identification in the Linear Tensile Range

Model 1 was developed to identify the effective mechanical composition of the BM from its experimentally measured tensile response in the linear range. An axisymmetric cylindrical representation was adopted, with a radius of 30 mm and a height of 90 mm. The domain was subdivided into interleaved regions assigned to HDPE, LDPE, and PP, whose relative thicknesses were parametrically varied to represent different constituent proportions. This layered configuration is a numerical idealization used to control the relative contribution of each polymer and does not imply that the actual recycled BM consists of physically separated polymer layers. The polymer fractions were systematically varied while maintaining a total composition of 100%, and the numerical response was subsequently compared with the experimental elastic modulus obtained in Phase II.

**Figure 5:**
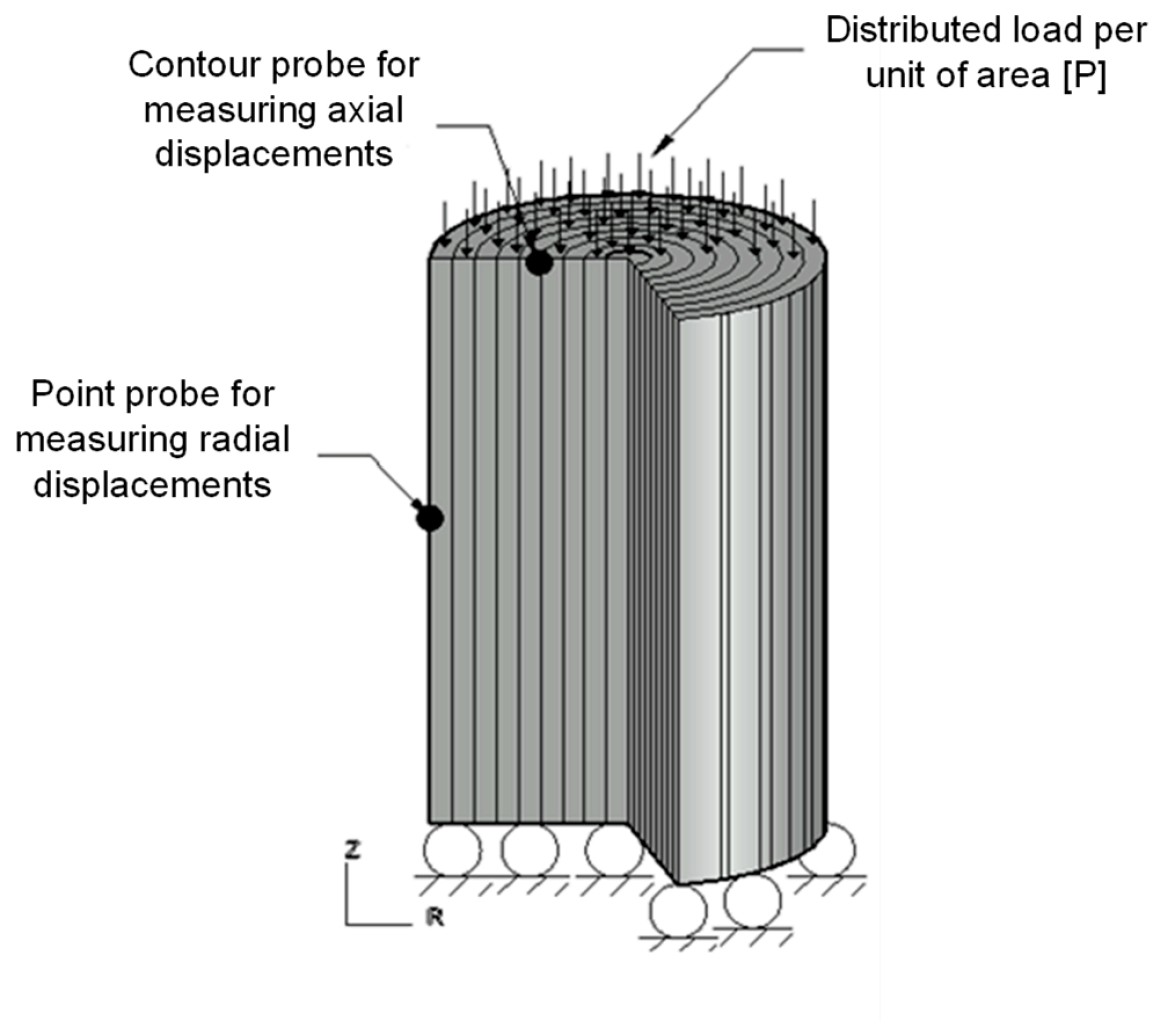
Model 1 configuration showing the uniformly distributed tensile pressure, axial and radial displacement probes, and roller-type constraint applied to the lower boundary.

A uniformly distributed tensile pressure ranging from 1 to 28 MPa was applied to the upper surface of the axisymmetric specimen, while a roller-type constraint was assigned to the lower boundary. Boundary and point probes were located at the upper and peripheral regions of the model to record axial (*Z*) and radial (*R*) displacements. The corresponding axial and radial strains were obtained from these displacements and used to determine the effective Young’s modulus and Poisson’s ratio for each trial composition. The loading, boundary conditions, and probe locations are illustrated in Figure 5.

The material properties assigned to the HDPE, LDPE, and PP domains were selected from published commercial and normative data and are summarized in Table 1. Because the reported elastic properties of recycled and commercial thermoplastics span relatively broad ranges, representative values within the cited ranges were adopted and subsequently kept constant during the parametric variation of the polymer fractions. Accordingly, the inverse-identification procedure should be interpreted as the determination of an effective mechanically equivalent composition for the adopted constituent properties, rather than as a unique chemical identification of the BM.

#### 2.3.3. Model 2: Verification Using the Experimental Tensile-Specimen Geometry

**Figure 6:**
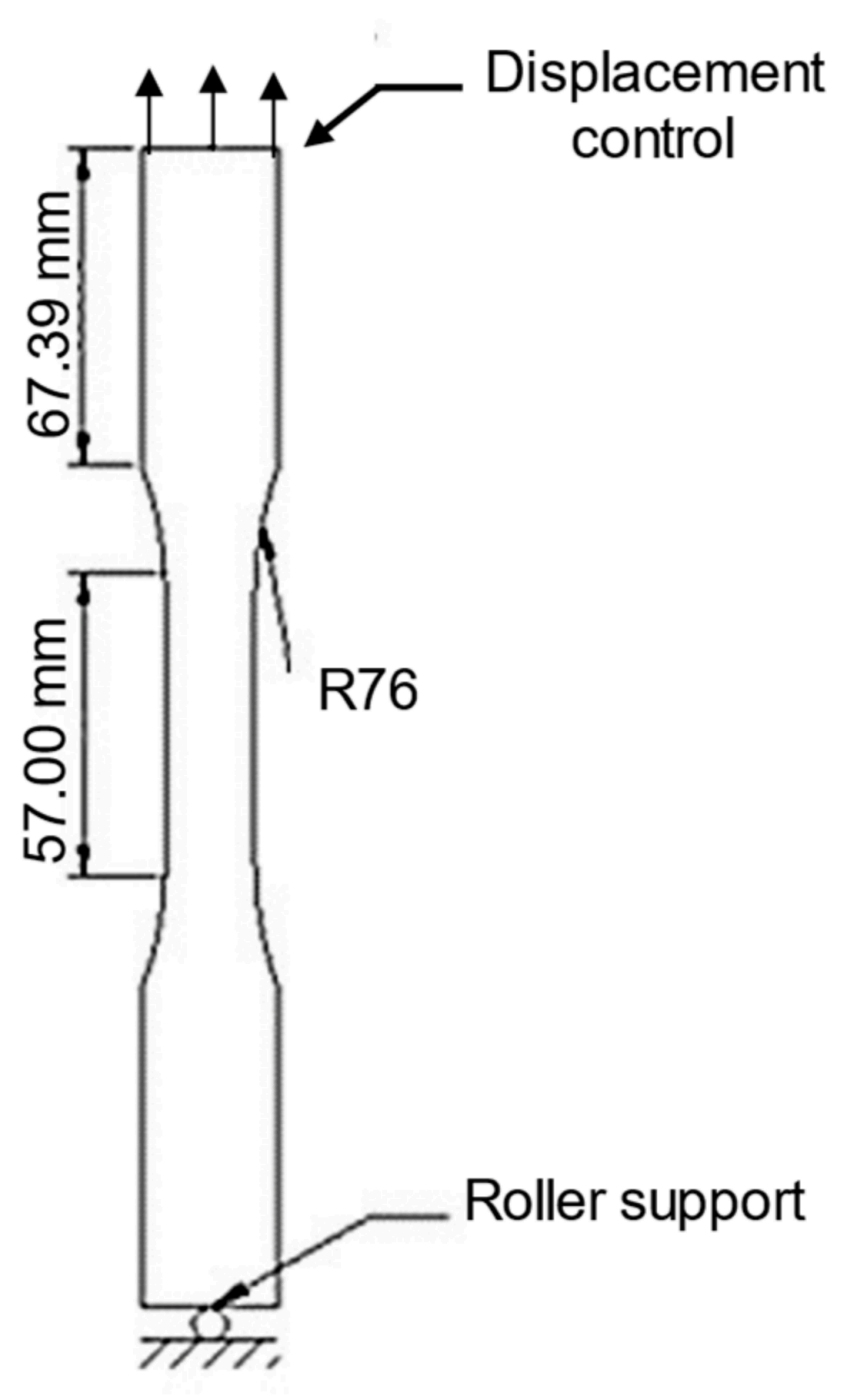
Model 2 geometry and boundary conditions used to verify the linear tensile response of the BM using a specimen representative of the experimental configuration.

After identifying the effective BM properties with Model 1, a second linear-elastic model was developed to verify the numerical response using a geometry representative of the experimentally tested tensile specimens. The BM was assigned *E* = 871.36 MPa and *ν* = 0.445, as obtained from the inverse-identification model. A prescribed axial displacement was applied to the upper boundary, while a roller-type constraint was prescribed at the lower boundary to reproduce the principal loading and restraint conditions of the laboratory test. The resulting tensile response was compared with the experimental response in the linear range, as discussed in Section 3.2.1. The model geometry and boundary conditions are shown in Figure 6.

#### 2.3.4. Model 3: Tensile Response in the Nonlinear Experimental Range

**Figure 7:**
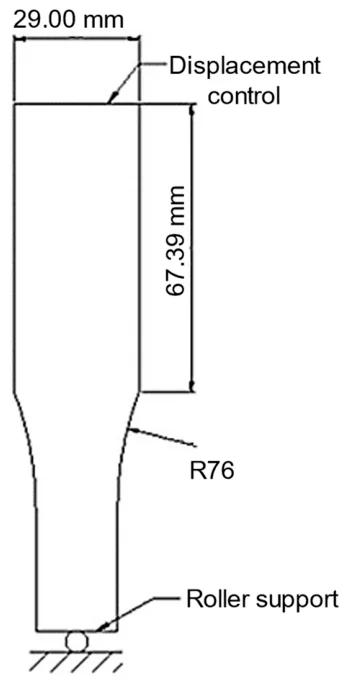
Model 3 two-dimensional half-specimen geometry and boundary conditions used to represent the BM tensile response in the nonlinear experimental range.

Model 3 was developed to examine the tensile response of the BM over the strain range corresponding to the experimentally observed nonlinear transition. To reduce computational cost, a two-dimensional planar model representing one-half of the tensile-specimen geometry used in Model 2 was adopted, taking advantage of the longitudinal symmetry of the specimen. A symmetry boundary condition was therefore prescribed along the corresponding cut boundary. The BM was assigned *E* = 871.36 MPa and *ν* = 0.445. Tensile loading was imposed through a prescribed axial displacement at the upper boundary. The corresponding axial stresses and strains were extracted from the numerical solution to characterize the simulated tensile response. For reference, Equation (5) describes the polynomial fit to the nonlinear transition region of the mean experimental tensile stress–strain curve. The axial strain, *ε_Z_*, was varied from 0 to 0.020 in increments of 0.0005, while a roller-type constraint was applied at the lower boundary. The model geometry and boundary conditions are shown in Figure 7.

(5) $$\sigma_{\exp,\mathrm{fit}}\left(\varepsilon_{Z}\right)=-15960\;\varepsilon_{Z}^{2}+921.55\;\varepsilon_{Z}+0.7457\;\left[\mathrm{MPa}\right]$$

where *ε_Z_* is the axial engineering strain [mm/mm], and *σ_exp_*_,*fit*_ is the tensile stress estimated from the polynomial fit to the mean experimental curve [MPa].

#### 2.3.5. Model 4: Numerical Optimization Through ABS Incorporation

Model 4 was developed to evaluate the effect of numerically incorporating ABS into the mechanically identified BM and to determine a composition with increased tensile stiffness. ABS and chlorinated polyethylene (CPE) were initially considered as potential polymer modifiers based on the literature; however, CPE was excluded from the numerical study because previous investigations reported incompatibility with LDPE [20]. Consequently, only ABS was retained for parametric analysis.

An axisymmetric cylindrical model analogous to that shown in Figure 5 for Model 1 was used, with interleaved numerical domains representing the BM and ABS. The BM composition was maintained internally at 26% HDPE, 36% LDPE, and 38% PP, while the ABS fraction was varied from 0 to 50% of the total material. As the ABS content increased, the BM fraction was reduced proportionally while preserving its internal HDPE/LDPE/PP ratio. The BM was assigned *E* = 871.36 MPa and *ν* = 0.445, whereas the ABS was modeled with *E* = 2300 MPa and *ν* = 0.39, based on PROSPECTOR [21]. The same tensile loading and displacement-probe strategy used in the preceding linear models was applied to determine the effective Young’s modulus and Poisson’s ratio for each composition. The optimal variant was defined as the composition providing the highest effective tensile modulus within the ABS range investigated; its thermal response was subsequently evaluated independently in Model 5.

#### 2.3.6. Model 5: Thermal Assessment of BM and CMo

**Figure 8:**
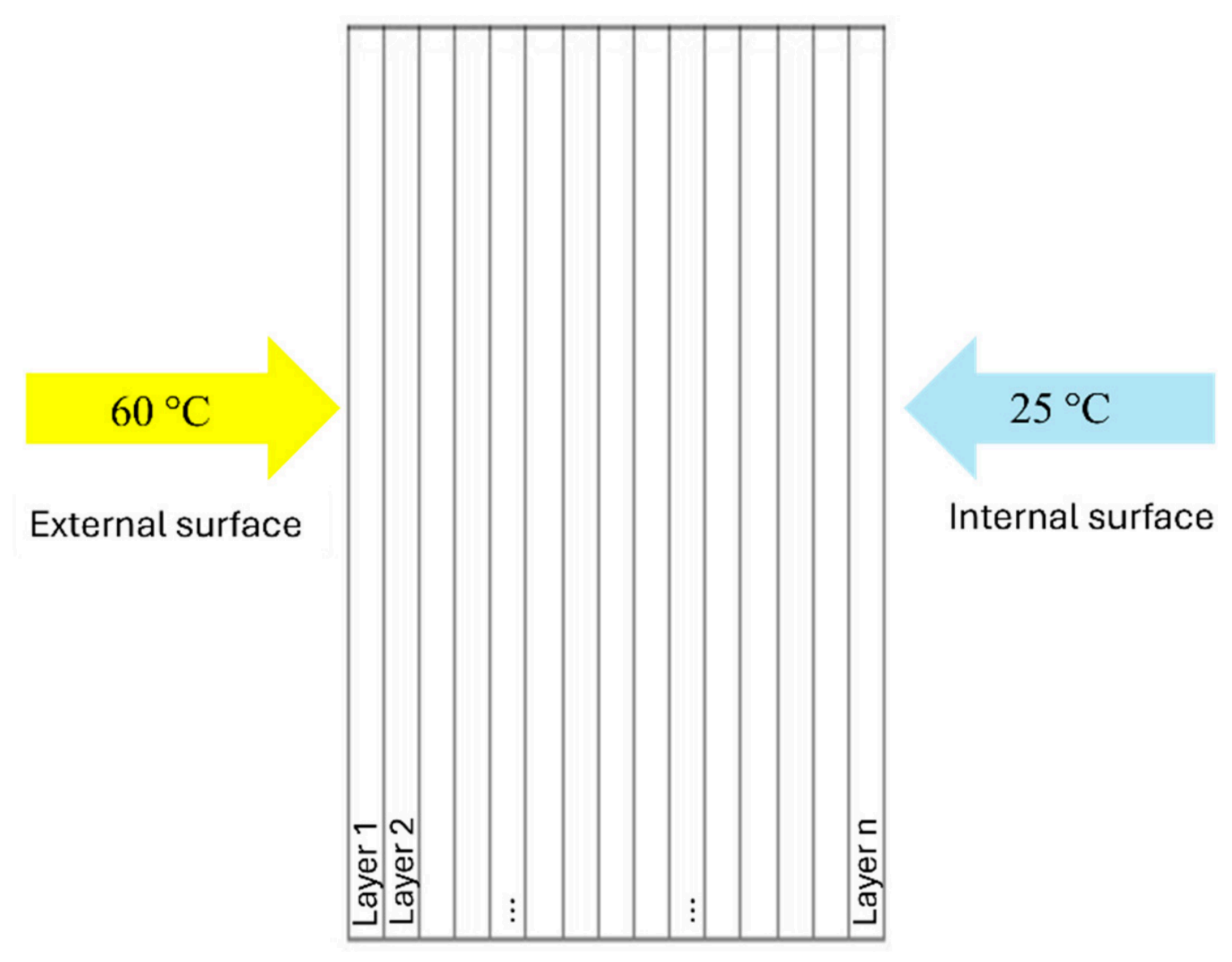
Model 5 plate geometry and thermal boundary conditions used to evaluate the temperature distribution of the BM and CMo.

Model 5 was developed to assess the thermal response of the BM and CMo under a prescribed through-thickness temperature gradient. A 150 × 150 × 100 mm plate was modeled using the Heat Transfer in Solids interface in COMSOL Multiphysics ^®^ 5.2a [16]. Temperatures of 60 °C and 25 °C were prescribed on two opposite faces of the plate, and the resulting temperature distribution through the specimen thickness was evaluated. The BM was represented using the effective composition identified in Model 1 (26% HDPE, 36% LDPE, and 38% PP), whereas the CMo incorporated 50% ABS while preserving the relative proportions of the BM constituents defined in Model 4. The model geometry and imposed thermal boundary conditions are shown in Figure 8.

The interleaved-domain configuration shown in Figure 8 is a numerical idealization used to assign the thermal properties of the constituent polymers according to their prescribed proportions. It does not represent physically separated polymer layers within the actual BM or CMo, whose microstructure corresponds to a heterogeneous recycled-polymer mixture. Accordingly, Model 5 should be interpreted as an effective representation of heat transfer through the two material formulations rather than as a direct simulation of their internal morphology. The analysis does not constitute a standardized fire-resistance test.

As an initial reference, each polymer was assigned independently to the entire plate to obtain its individual thermal response. The simulations were then performed using the BM composition identified in Model 1 and the CMo composition defined in Model 4. The thermal conductivity values assigned to the constituent polymers are summarized in Table 2. The thermal result reported in Section 3.2.2 is limited to the BM temperature distribution.

#### 2.3.7. Model 6: Study of Mechanical Behavior Under Compression

A cylindrical specimen was modeled with the same interleaved-domain distribution as described in Model 1 (Section 2.3.2). The chosen geometry was selected to promote a radially symmetric stress distribution across the specimen and avoid geometric stress concentrations. Fixed polymer proportions were assigned to represent both the BM (26% HDPE, 36% LDPE, and 38% PP) and the CMo (13% HDPE, 18% LDPE, 19% PP, and 50% ABS). The compressive elastic moduli used in this model were selected based on literature data [22,23,24,25] and are summarized in Table 3.

The compressive response was evaluated by applying a uniformly distributed pressure, *P*, ranging from 1 to 28 MPa in increments of 9 MPa. Two boundary probes were used to record the radial and longitudinal displacements, from which the corresponding strains were calculated. A stationary study with a parametric sweep of *P* was used, without geometric nonlinearity. The automatic physics-controlled meshing procedure described in Section 2.3.1 was retained for this model.

After the simulations were completed, the compressive behavior of the CMo was compared with that of a 28 MPa concrete reference to assess its relative stiffness and deformation response and its potential use in compression-dominated structural elements, such as columns.

### 2.4. Phase IV: Structural and Environmental Feasibility Assessment

#### 2.4.1. Single-Family Home Design

**Figure 9:**
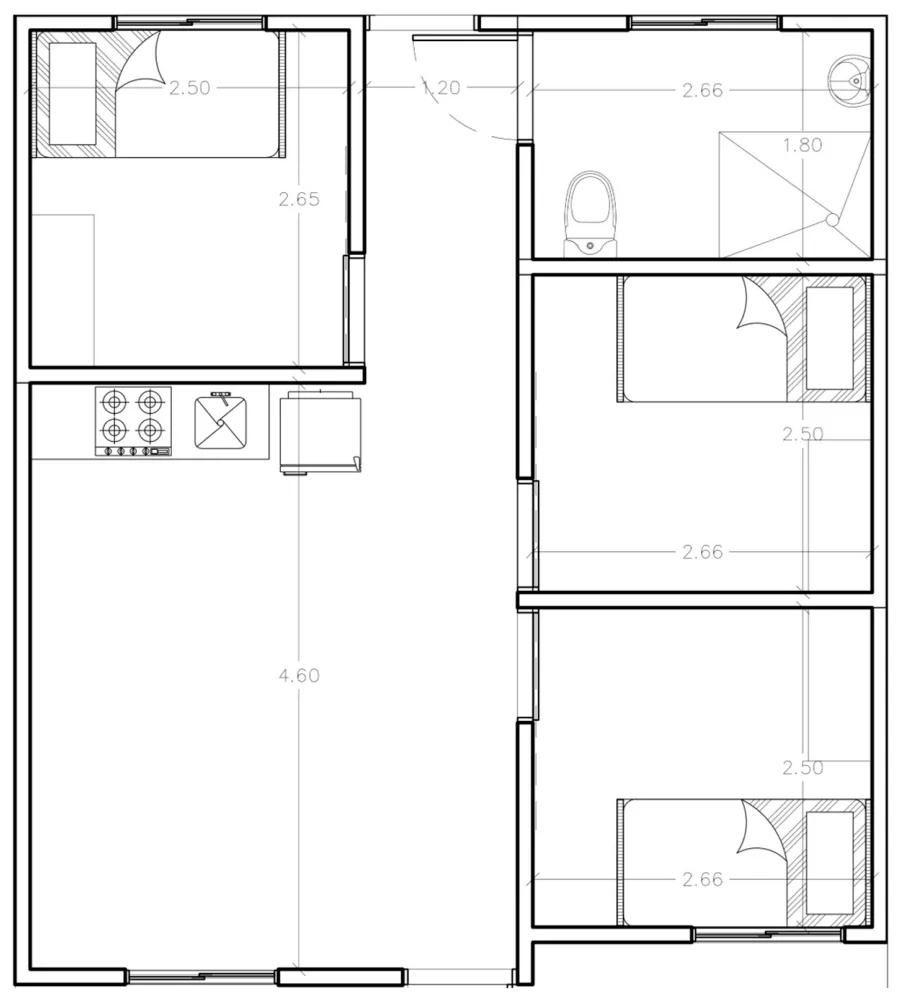
Architectural floor plan of the single-family residential case study (dimensions in meters).

Once the tensile and compressive behavior of the CMo in the linear range had been numerically estimated, a preliminary structural feasibility assessment of a single-story residential building was carried out (Figure 9). The house has a surface area of 85 m^2^, which is consistent with dimensions commonly accepted for low-income housing projects in Colombia [26]. The design includes reinforced-concrete foundation beams with upper and lower longitudinal reinforcement and confinement stirrups along their entire length, as outlined in ACI PRC-314-16 [27]. The foundation slab is 0.10 m thick, considering the proposed reinforcement and the applicable durability and minimum-cover requirements. For design purposes, it was assumed that the supporting soil beneath the slab would be compacted with selected granular material to provide adequate bearing capacity and uniform load distribution.

**Figure 10:**
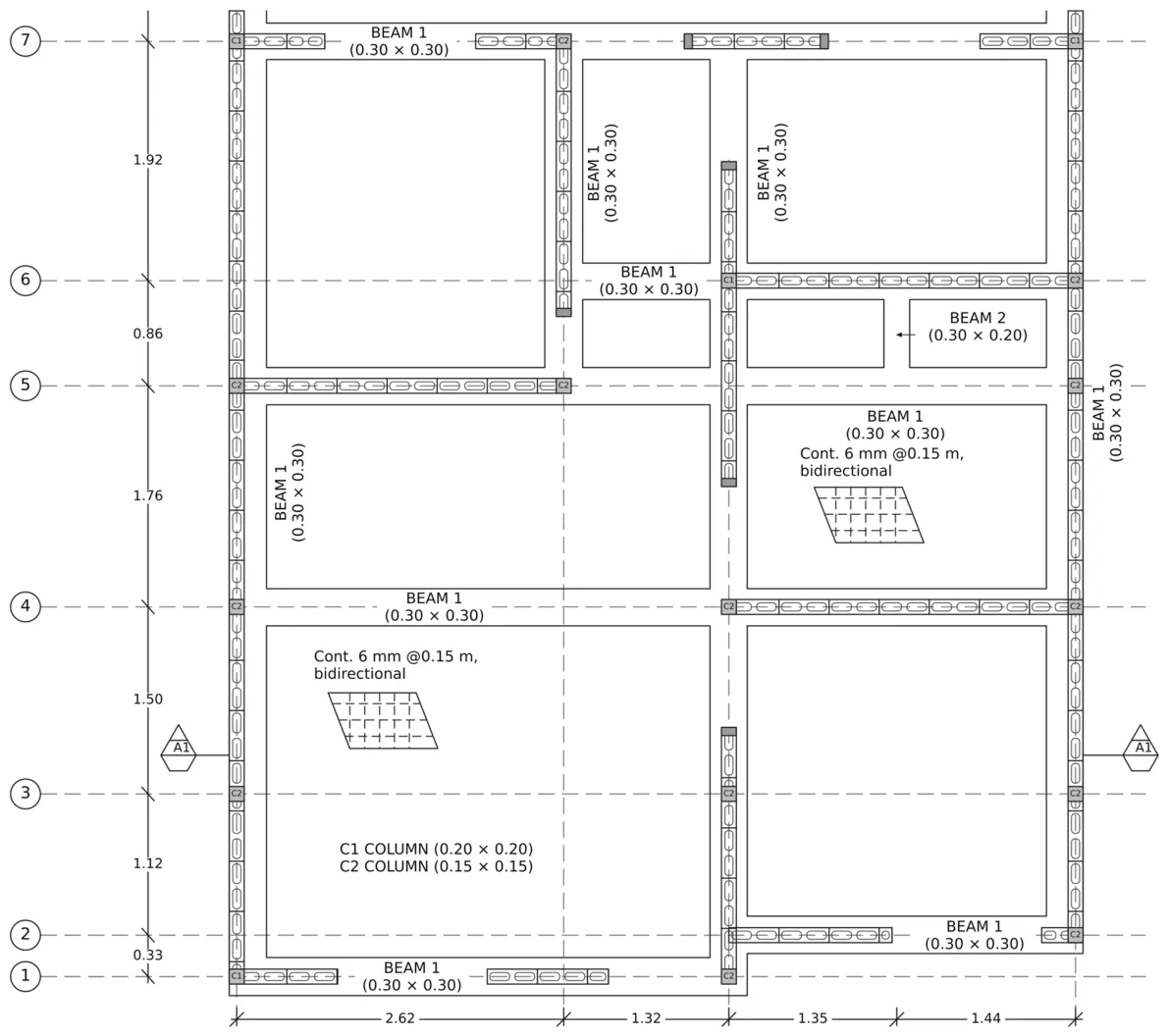
Foundation plan and column placement. All dimensions are in [m] unless otherwise indicated.

The structural columns, modeled entirely in CMo, were positioned to provide a regular load-transfer arrangement within the adopted building configuration. Based on the tensile and compressive properties adopted for the CMo and considering the guidelines established in section E.4.3.3 of the Colombian Earthquake-Resistant Construction Regulations (NSR-10) [28], a conservative maximum column spacing of 3 m was adopted. Although this provision applies to reinforced-concrete columns in confined-masonry houses, it was used here only as a geometric reference for the preliminary feasibility assessment. This decision was also consistent with the proposed floor-plan configuration and the 0.10 m wall thickness selected from available industrial proposals [29]. A column height of 2.5 m was adopted for the case study. These geometric values should not be interpreted as validated design limits for CMo construction. Figure 10 shows the foundation floor plan of the house, excluding the patio area, together with the locations of the foundation beams and columns, represented by gray squares. The structural model of the building frame was developed using ETABS^®^ 2015.

To estimate the compressive response of the CMo, a bilinear stress–strain model was developed for each polymer, incorporating a post-yield slope based on the input parameters. The stress–strain curve of the CMo was approximated through a section analysis, assuming a unit area and strain compatibility among all polymers. The tensile response was obtained using the same methodology as for compression. Since the yield stress and strain of the CMo had not been experimentally determined, the bilinear models used yield and ultimate stress–strain parameters adopted for the individual polymers. The compression parameters were obtained from previous studies [22,23,24,25], whereas the tensile parameters were obtained from MatWeb [30] as indicated in Table 4.

*E_s_* is the initial elastic modulus, *f_y_* and *ε_y_* are the yield stress and strain, *f_u_* and *ε_u_* are the ultimate stress and strain, and *E_h_* is the post-yield tangent modulus. The calculated parameters satisfy *ε_y_* = *f_y_*/*E_s_* and *E_h_* = (*f_u_* − *f_y_*)/(*ε_u_* − *ε_y_*). The negative value of *E_h_* for PP under tension represents post-yield softening because the adopted ultimate stress is lower than the yield stress. The CMo proportions were represented as proportional areas in the unit-section analysis.

It is important to clarify that these bilinear models were used to estimate the mechanical behavior of the CMo under tensile and compressive loads rather than being directly assigned as complete constitutive curves in ETABS^®^. The stress–strain curves obtained from the section analysis therefore provide theoretical estimates of the CMo response. Because only the BM, and not the CMo, was experimentally tested, the estimated CMo yield and ultimate properties should not be interpreted as experimentally validated values. The properties implemented in the preliminary ETABS^®^ model included the modulus of elasticity, Poisson’s ratio, density, and the estimated yield and ultimate stress–strain parameters in compression and tension.

The input parameters for the structural model are summarized in Table 5. A gravity-load combination of 1.2*D* + 1.6*L*, where *D* and *L* denote the dead and live loads, respectively, was included in the analysis. Seismic effects were also considered, and the parameters presented in Table 5 were used to define the design spectrum. The load combinations established in NSR-10 [28] were adopted for this preliminary structural assessment. Because the energy-dissipation capacity of the CMo structural system has not been experimentally determined, a response modification coefficient, *R*, of 1.0 was conservatively assumed.

The section list used in ETABS^®^ 2015 was established through preliminary member sizing. Square sections of 0.15 m × 0.15 m and 0.20 m × 0.20 m were considered for the columns, whereas a rectangular section of 0.15 m × 0.10 m was considered for the lintel beams and roof purlins. The slenderness check was performed for the smallest column section. Using an effective length factor (*K*) of 1.0, an unsupported length (*L*) of 2.5 m, and a radius of gyration (*r*) of 43.3 mm, the resulting slenderness ratio (*KL*/*r*) was 57.73, which is below the limiting value of 200 adopted from McCormac and Csernak [31]. This calculation was used as a preliminary screening of member slenderness. For the roof purlins, the allowable deflection was set at *L*/240, in accordance with the International Building Code [32].

**Figure 11:**
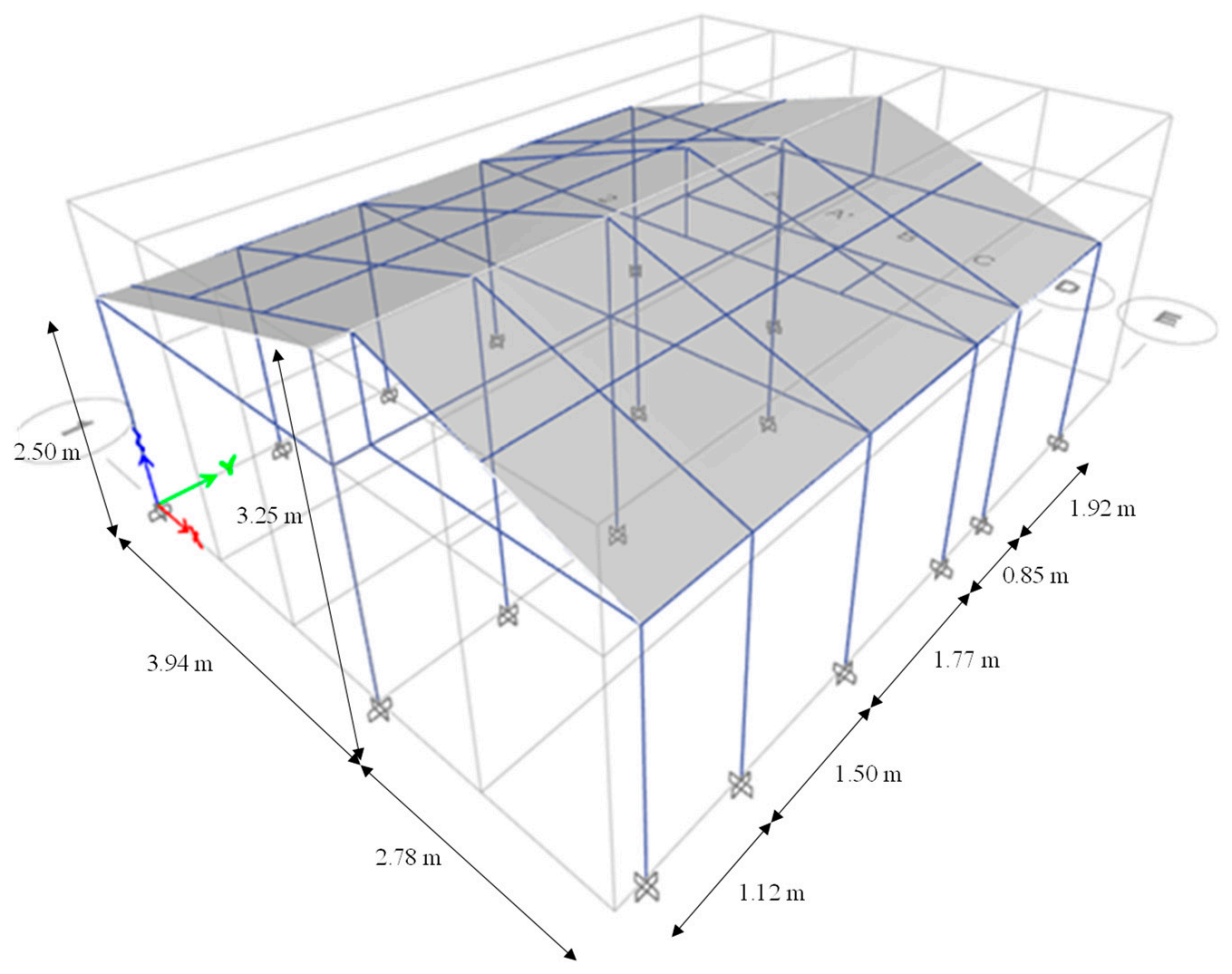
Geometric configuration of the portal-frame model developed in ETABS ^®^.

Figure 11 shows the geometric configuration of the portal-frame model developed in ETABS^®^ 2015. The capacity-reduction factors assigned in the model design preferences were 0.80 for flexure, compression, and tensile yielding; 0.55 for tensile failure; and 0.70 for shear and torsion. These factors were adopted as provisional inputs for the preliminary structural assessment and were not calibrated against experimental results from CMo structural members. Consequently, they should not be interpreted as validated design factors and require experimental verification in future work.

Finally, story drifts were evaluated in ETABS^®^ under seismic and wind loading conditions considered in the study. A wind pressure of 0.4 kN/m^2^, corresponding to the minimum value specified in NSR-10 [28], was adopted for the preliminary analysis. For the seismic assessment, a maximum story-drift ratio of 1.0% was used as a reference criterion based on the NSR-10 limit for frame systems [28]. Because structural systems constructed with CMo are not covered by this regulation, the drift evaluation should be interpreted as a preliminary feasibility check rather than a demonstration of regulatory compliance.

#### 2.4.2. Comparative CO_2_ Emissions Assessment

This phase estimated CO_2_ emissions associated with the quantities of materials required for the main structural components of the proposed housing system. The CMo alternative, composed of 13% HDPE, 18% LDPE, 19% PP, and 50% ABS, was compared with a conventional alternative comprising reinforced-concrete beams and columns and steel roof purlins. Both alternatives corresponded to the same architectural configuration and were evaluated under the same load demands. The numerically selected CMo composition from Phase III was retained and was not reoptimized during the environmental assessment.

Emission factors were obtained from the literature and environmental declarations for cement, water, sand, gravel, reinforcing steel, structural steel tubing, HDPE, LDPE, PP, and ABS. Table 6 summarizes the factors adopted in the comparative assessment. For each structural alternative, the mass of each constituent material was calculated from the corresponding element volumes, material proportions, and densities. Total emissions were then estimated as the sum of the mass of each material multiplied by its respective emission factor. Factors reported as CO_2_ for the concrete constituents and in-plant concrete production were used as approximations of CO_2_-equivalent emission factors. No additional project-specific transport emissions were calculated because reliable information on transportation routes, distances, and modes was unavailable; transport contributions already included in the source datasets were retained. Accordingly, the results should be interpreted as a preliminary inventory-based comparison using sources with differing system boundaries, rather than as a complete life-cycle assessment.

Product-specific emission factors were adopted for reinforcing bars and structural steel tubing. The Latin American average reported by worldsteel was used for reinforcement [33], whereas the ACESCO environmental product declaration was used for the roof purlins, assuming ungalvanized structural steel tubing [34]. For both steel products, the A1–A3 total global warming potential was used, excluding end-of-life modules and module D.

**Table 6:** Literature-based emission factors adopted for the comparative assessment.

Material	Adopted Emission Factor [kgCO_2_-eq/kg Material]
Recycled PP (RPP)	0.10 ^(1)^
Recycled LDPE (RLDPE)	0.60 ^(1)^
Recycled HDPE (RHDPE)	0.63 ^(2)^
Recycled ABS (rABS)	0.62 ^(3)^
Ordinary Portland Cement (OPC)	0.931 ^(4)^
Sand	2.6 × 10^−3 (4)^
Gravel	7.5 × 10^−3 (4)^
Water	1.96 × 10^−4 (4)^
Reinforcing steel—Latin American average	1.34 ^(5)^
Ungalvanized structural steel tubing—Colombia	2.81 ^(6)^

^(1)^ AlMa’adeed et al. [35]; ^(2)^ Bataineh [36]; ^(3)^ Da Silva et al. [37]; ^(4)^ Yang et al. [38]; ^(5)^ World Steel Association [33]; ^(6)^ ACESCO [34].

## 3. Results and Discussion

### 3.1. Experimental Characterization Results

#### 3.1.1. Tensile Test

**Figure 12:**
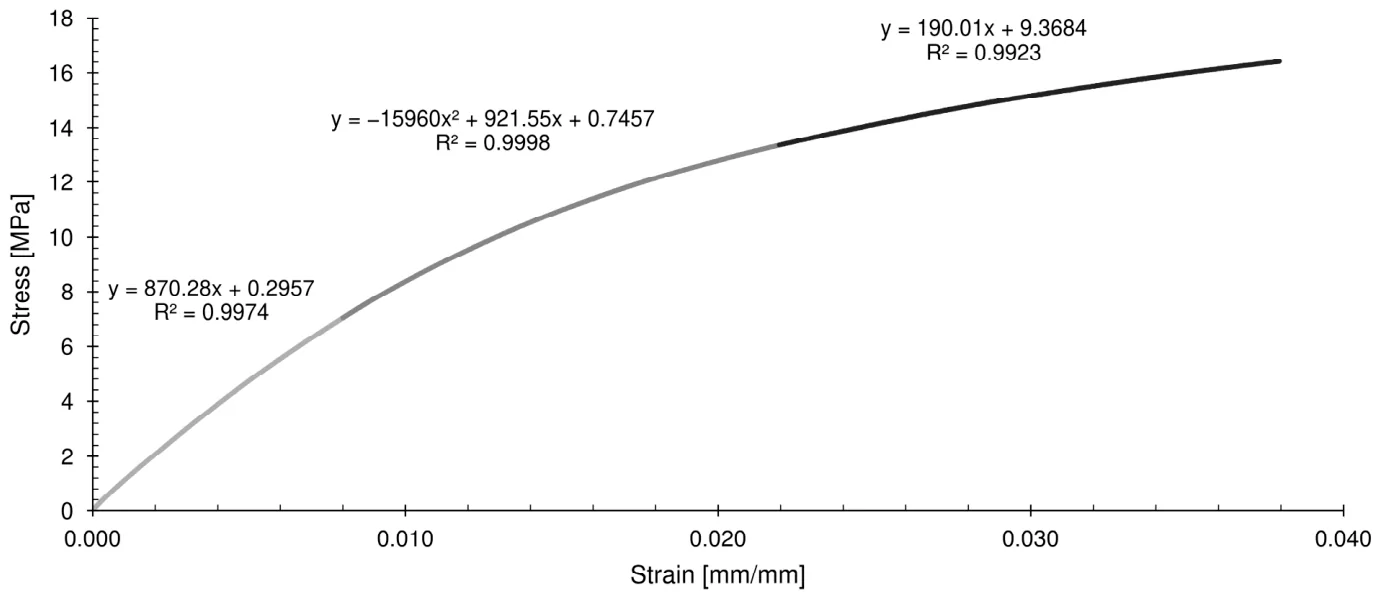
Mean experimental tensile stress–strain curve of the BM and regression fits for the initial linear, nonlinear transition, and final response regions.

Figure 12 presents the mean experimental tensile stress–strain curve of the BM, obtained by averaging the recorded stress and strain values at corresponding data acquisition points for the four valid specimens described in Section 2.2.1; therefore, it does not represent the response of a single specimen. Three regression expressions were fitted to different regions of the mean curve. The initial approximately linear region is described by *σ* = 870.28*ε* + 0.2957 (R^2^ = 0.9974), whose slope gives an experimental elastic modulus of 870.28 MPa. The nonlinear transition region is represented by *σ* = −15960*ε*^2^ + 921.55*ε* + 0.7457 (R^2^ = 0.9998). The final fitted region is described by *σ* = 190.01*ε* + 9.3684 (R^2^ = 0.9923).

The reduction in the fitted slope from 870.28 MPa in the initial region to 190.01 MPa in the final region—approximately 78%—indicates a substantial decrease in tangent stiffness, although the tensile stress continued to increase. The R^2^ values indicate close agreement between each regression and the experimental data within its corresponding fitted interval. The four valid specimens reached a mean maximum tensile stress of 17.35 MPa (sample standard deviation: 0.96 MPa; range: 15.96–18.13 MPa). Post-test measurements, calculated from the initial and final gauge lengths after rupture, yielded elongation percentages ranging from 0.70% to 1.75% and reductions in cross-sectional areas ranging from 0.05% to 6.35%. These measurements indicate limited permanent deformation before rupture under the tested conditions.

The three fitted expressions are empirical descriptors of distinct regions of the mean experimental curve rather than constitutive laws, and each fulfils a specific role within the methodology. The slope of the initial linear fit provides the experimental elastic modulus used as the target quantity for the inverse identification of the effective BM composition (Models 1 and 2, Section 2.3); the fit of the nonlinear transition region provides the reference expression for the strain range examined in Model 3 (Equation (5)); and the final fit characterizes the response at larger strains. The quadratic expression is interpolative within its fitted interval only: its negative leading coefficient would predict a stress maximum at a strain of approximately 0.029 mm/mm, beyond the fitted range, and it should therefore not be extrapolated.

Beyond their numerical values, the three regions describe a response that differs from that typically reported for the individual constituent polymers. The tangent stiffness decreases progressively from 870.28 MPa to 190.01 MPa, a reduction of approximately 78%, without a distinct yield point or the subsequent stress plateau associated with cold drawing in semicrystalline polyolefins. This gradual loss of stiffness, together with the limited permanent deformation measured after rupture, indicates a macroscopically brittle response rather than the extensive ductile deformation characteristic of PP, HDPE, and LDPE individually. Reprocessing is known to reduce the ductility of polyolefins. For isotactic polypropylene subjected to six reprocessing cycles, the elongation at break decreases from approximately 66% to 52% and the fracture toughness decreases, while the elastic modulus and yield stress increase as a consequence of a higher degree of crystallinity [5]. Embrittlement of recycled polypropylene has likewise been reported through a pronounced reduction in impact strength, even though the tensile yield strength and elastic modulus remain essentially unchanged [4]. The elongation values measured for the BM are, however, one to two orders of magnitude below those reported for reprocessed polypropylene. Reprocessing-induced degradation alone therefore cannot account for the response observed here, which supports the interpretation that the fracture behavior of the BM is governed primarily by its heterogeneous blended nature and by the internal defects present in the material as supplied.

Two characteristics of the BM are consistent with this behavior. First, it is a blend of recycled polyolefins processed without compatibilizers, and the mechanical response of such blends is known to depend strongly on the proportions and interaction of the constituent phases [6]. Second, internal porosity was observed in the supplied sections during cutting (Figure 3), which reduces the effective load-bearing area and introduces stress concentrators; the influence of processing conditions on internal defects and on the resulting tensile performance of recycled thermoplastics has been documented previously [8]. Under these conditions, the progressive reduction in tangent stiffness is more consistent with the accumulation of distributed damage at phase boundaries and around internal voids than with matrix-controlled yielding of a homogeneous polymer. It must be emphasized that no microstructural or fractographic characterization (e.g., scanning electron microscopy, differential scanning calorimetry, or infrared spectroscopy) was performed in this study. These interpretations are therefore consistent with the observed macroscopic response but are not experimentally demonstrated, and they are proposed as hypotheses for subsequent investigation.

Despite this defect-sensitive behavior, the scatter among the four valid specimens was limited, with a sample standard deviation of 0.96 MPa on a mean maximum tensile stress of 17.35 MPa (coefficient of variation of approximately 5.5%), indicating that the response of the material as supplied was reproducible within the tested group. Accordingly, the value of 870.28 MPa should be interpreted as an effective modulus of this specific recycled stream in its as-received processing condition, including its internal defect state, rather than as an intrinsic property of a PP/HDPE/LDPE blend of the same nominal composition. This interpretation is appropriate for the purpose of the present study, since the numerical models were calibrated to reproduce the material actually tested, but it limits the direct transferability of the reported value to recycled streams processed under different conditions.

#### 3.1.2. Compression Test

Table 7 summarizes the maximum loads and corresponding compressive stresses recorded for the three BM specimens, giving an average stress at test termination of 18.74 MPa. As described in Section 2.2.2, none of the specimens reached physical failure; therefore, this value represents the average maximum sustained compressive stress rather than the measured compressive strength of the BM. Considering the experimentally determined BM bulk density of 970 kg/m^3^, the specified concrete compressive strength of 28 MPa, and the reference reinforced-concrete density of 2400 kg/m^3^, the sustained compressive-to-density ratio of the BM was at least 1.66 times the corresponding specified strength-to-density ratio of the reference concrete. Additionally, for equal volumes, the concrete element would have approximately 2.47 times the mass of the corresponding BM element. These results indicate favorable specific compressive performance of the BM, although its ultimate compressive strength was not determined. The specimens also exhibited visible deformation recovery after unloading; however, this observation was qualitative.

Because all three tests were terminated at a comparable preset load of approximately 76.6 kN rather than at failure, the narrow range of the reported stresses (18.6–18.8 MPa) reflects the small differences in the measured loaded areas rather than the variability of a material property. The reported average of 18.74 MPa should therefore be interpreted as a lower bound on the compressive resistance of the BM under the adopted test configuration, and not as a compressive strength, a yield stress, or a measure of experimental scatter.

The compressive and tensile responses of the BM differ markedly. Under tension, the material ruptured at a mean maximum stress of 17.35 MPa with elongation after fracture between 0.70% and 1.75%, whereas under compression, it sustained a comparable stress level without reaching failure and with substantial, visibly recoverable deformation. This asymmetry is consistent with the pressure-dependent yielding generally exhibited by polymers, for which the yield stress under compression exceeds that under tension, and with the defect population discussed in Section 3.1.1: internal voids and poorly bonded interfaces between the recycled polyolefin phases act as crack initiators under tensile loading, whereas under compressive loading, these same features tend to close, partially restoring load transfer across them. The combination of brittle tensile fracture and ductile, recoverable compressive behavior is therefore attributable to the heterogeneous and defect-containing nature of the material as supplied, rather than to an intrinsic difference between the constituent polymers.

From the standpoint of structural application, the favorable specific compressive performance reported above should be interpreted with care, since resistance per unit density is not the governing criterion for the members considered in this study. The numerically predicted effective compressive modulus of the BM (756.15 MPa, Section 3.2.3) is more than an order of magnitude below the elastic modulus typically associated with a 28 MPa structural concrete, so serviceability deflections, axial shortening, and lateral stiffness—rather than compressive resistance—govern the dimensioning of the proposed elements. This is consistent with the structural assessment reported in Section 3.3, where insufficient lateral stiffness under seismic loading, and not insufficient compressive capacity, was identified as the limiting condition.

Finally, the limitations of the adapted test configuration should be acknowledged. The 50 mm cubic geometry has an aspect ratio of unity, so restraint at the loading platens introduces a degree of lateral confinement; this effect is accepted for hydraulic-cement mortars but is more pronounced for a highly deformable polymer. In addition, axial strain was not recorded during the compression tests, so no experimental compressive stress–strain curve or compressive modulus could be derived from them; the compressive moduli adopted for the subsequent numerical models were obtained from literature stress–strain curves (Table 3). Compressive characterization using instrumented strain measurement, lubricated platens, and controlled strain rates is therefore identified as necessary future work.

### 3.2. Numerical Modeling and Material Optimization

#### 3.2.1. Models 1–4: Tensile Response and Material Optimization

**Figure 13:**
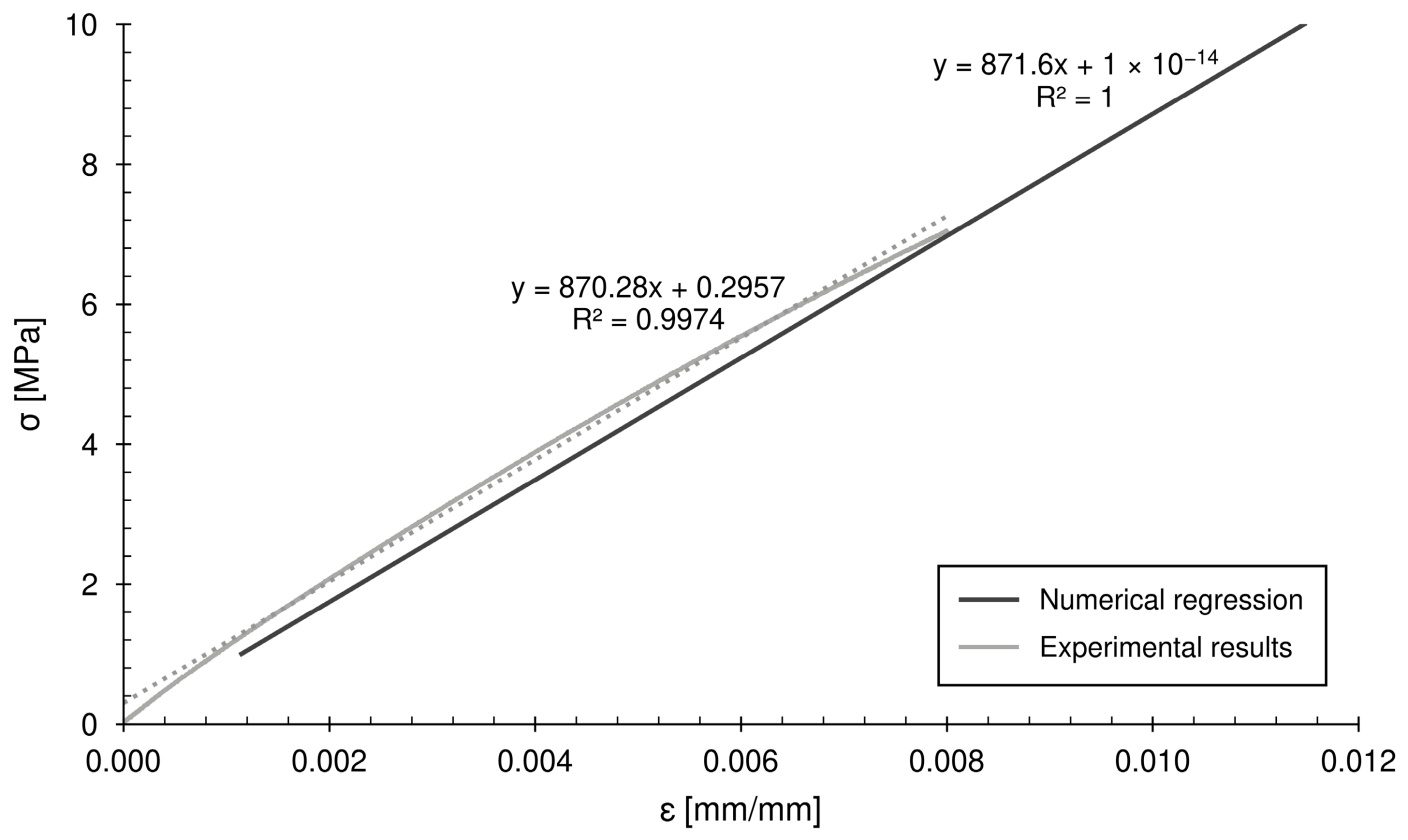
Experimental and numerical tensile stress–strain regressions in the linear range for the BM (26% HDPE, 36% LDPE, and 38% PP).

Model 1 used axial and radial displacement probes to evaluate the response to variations in the applied load and in the HDPE, LDPE, and PP proportions assigned to the idealized polymer domains. Through the inverse identification procedure described in Section 2.3.2, the composition providing the closest match to the experimental elastic modulus was 26% HDPE, 36% LDPE, and 38% PP. As clarified in Section 2.1, these proportions define an effective mechanically identified BM composition rather than a chemically measured formulation. The corresponding effective properties, *E* = 871.36 MPa and *ν* = 0.445, were subsequently assigned to Model 2, which reproduced the experimental specimen geometry to verify the linear response. Figure 13 compares the experimental and numerical regressions in the initial linear range. The experimental data followed *σ* = 870.28*ε* + 0.2957 (*R*^2^ = 0.9974), whereas the numerical results followed *σ* = 871.6*ε* + 1 × 10^−14^ (*R*^2^ = 1.0000). The slopes differed by approximately 0.15%, indicating close agreement in the fitted linear response. Because the effective material properties assigned to Model 2 were obtained from the preceding inverse-identification stage, this comparison should be interpreted as a consistency check rather than as an independent validation.

Model 1 identified an effective Young’s modulus of 871.36 MPa for the BM, which differed by 0.124% from the experimental value of 870.28 MPa. The effective properties identified in Model 1 were subsequently assigned to Models 2 and 3; therefore, these models should not be interpreted as independent estimates of Young’s modulus. Instead, Model 2 examined whether the linear response was maintained when the experimental tensile-specimen geometry was represented, whereas Model 3 extended the numerical assessment to the nonlinear experimental range.

**Figure 14:**
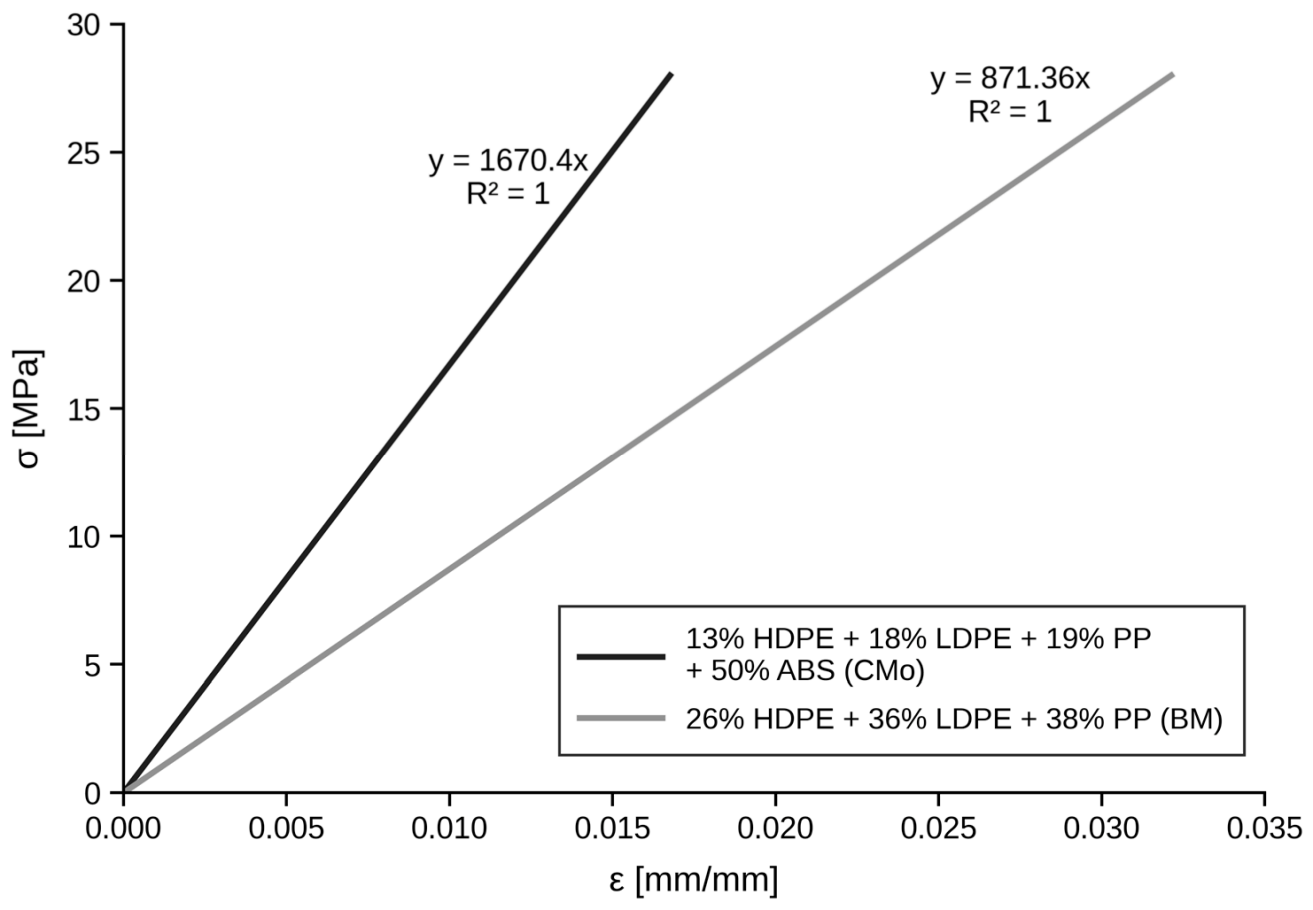
Numerically predicted tensile stress–strain responses of the BM (26% HDPE, 36% LDPE, and 38% PP) and the numerically optimized composite, CMo (13% HDPE, 18% LDPE, 19% PP, 50% ABS).

In Model 4, the ABS content was varied from 0 to 50% while the relative proportions of HDPE, LDPE, and PP within the BM were maintained. At the highest ABS content considered, Young’s modulus increased by 91.7%, from 871.36 MPa to 1670.4 MPa (Figure 14), while Poisson’s ratio decreased from 0.445 to 0.411. The latter result indicates a reduction in transverse strain relative to axial strain and should not be interpreted independently as evidence of increased deformation capacity. Within the investigated range, the composition containing 50% ABS, 19% PP, 13% HDPE, and 18% LDPE was therefore selected as the numerically optimized composite (CMo) for the subsequent analyses.

#### 3.2.2. Model 5: Temperature Distribution in the BM

**Figure 15:**
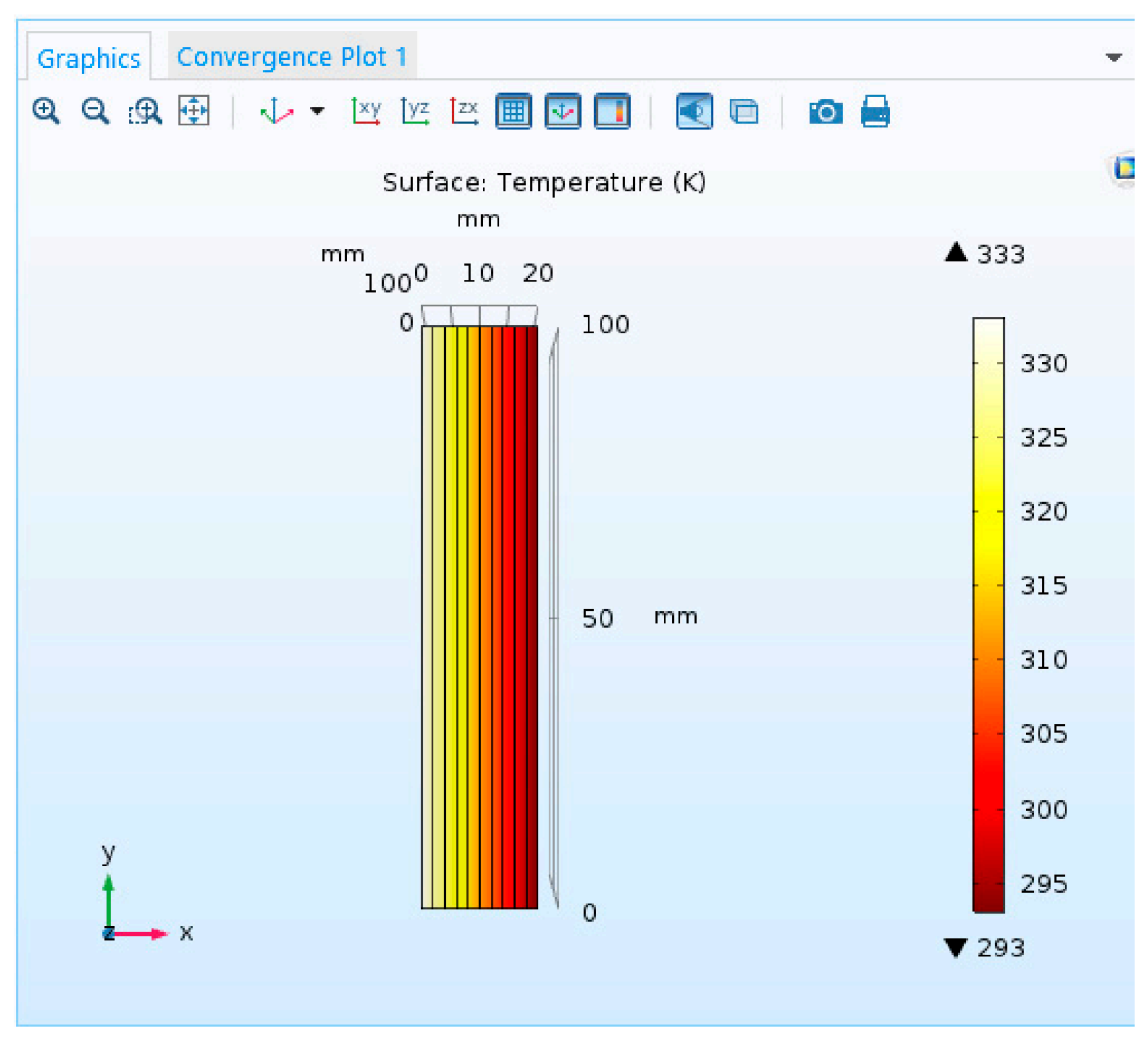
Numerically predicted temperature distribution in the BM represented by assigned material domains (26% HDPE, 36% LDPE and 38% PP) under boundary temperatures of 60 °C and 25 °C.

Figure 15 illustrates the numerically predicted temperature distribution within the BM for boundary temperatures of 60 °C and 25 °C applied to opposite faces. Although the same geometry and domain-assignment procedure were defined for the CMo, Figure 15 presents only the BM temperature field and does not establish a quantitative BM–CMo thermal comparison. The temperature decreases from the hotter face toward the colder face according to the spatial distribution of the constituent domains and their assigned thermal conductivities. This result represents the effective thermal response of the simplified domain configuration described in Section 2.3.6.

The interleaved-domain arrangement shown in Figure 8 and Figure 15 is a numerical idealization and should not be interpreted as the physical microstructure of the manufactured BM or as implying that the proposed CMo would consist of discrete polymer layers. Instead, both materials are represented as heterogeneous polymer mixtures through assigned material domains.

#### 3.2.3. Model 6: Study of Mechanical Behavior Under Compression

**Figure 16:**
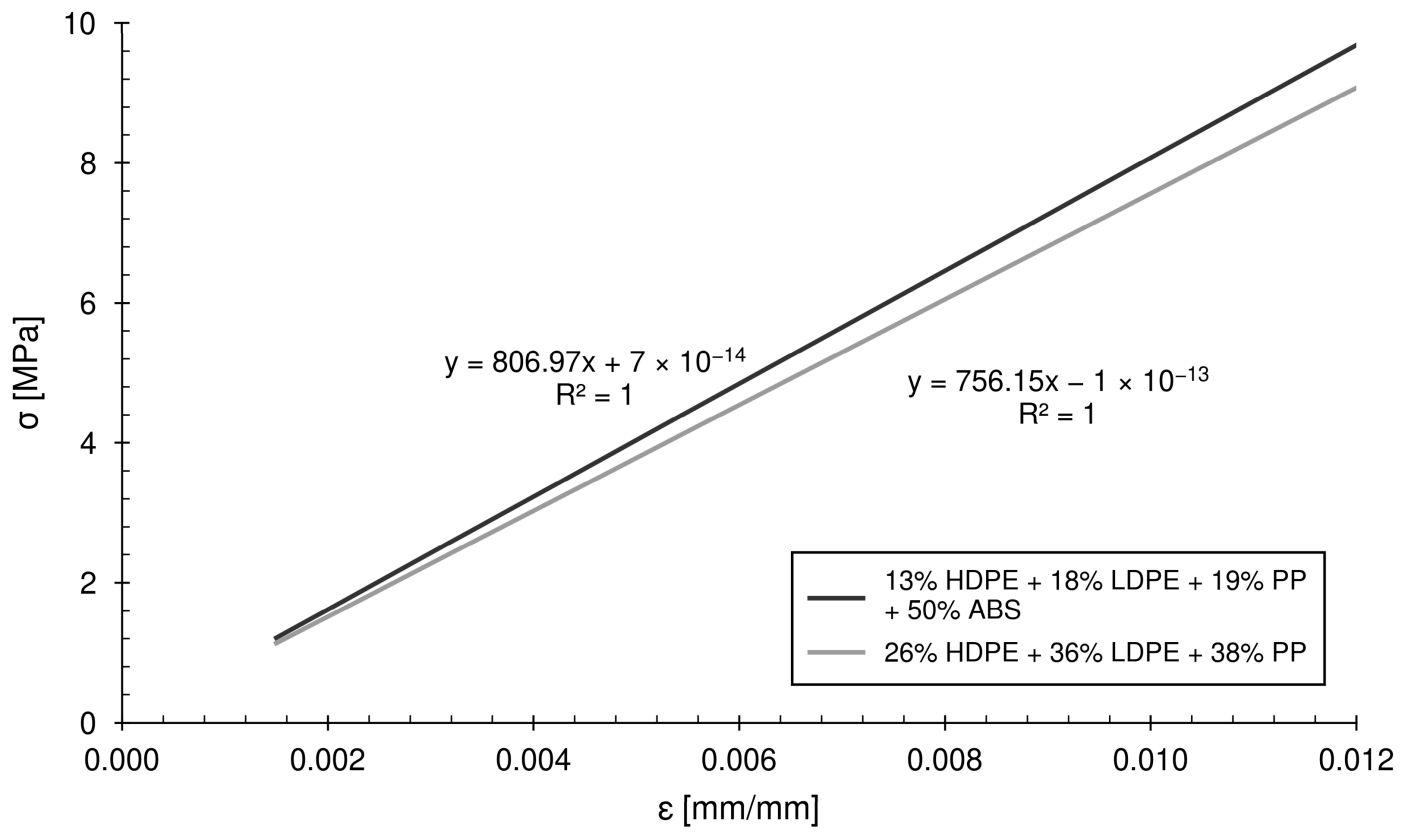
Numerically predicted compressive stress–strain curves and linear regressions for the BM (assigned domain fractions: 26% HDPE, 36% LDPE, and 38% PP) and the CMo (13% HDPE, 18% LDPE, 19% PP, and 50% ABS).

Model 6 allowed comparison of the compressive response of the BM and CMo. As shown in Figure 16, incorporating an assigned ABS fraction of 50% increased the numerically predicted effective compressive modulus from 756.15 MPa for the BM to 806.97 MPa for the CMo, corresponding to an increase of approximately 6.7%. This increase indicates that the CMo develops lower longitudinal strains than the BM under the same applied compressive stress.

**Figure 17:**
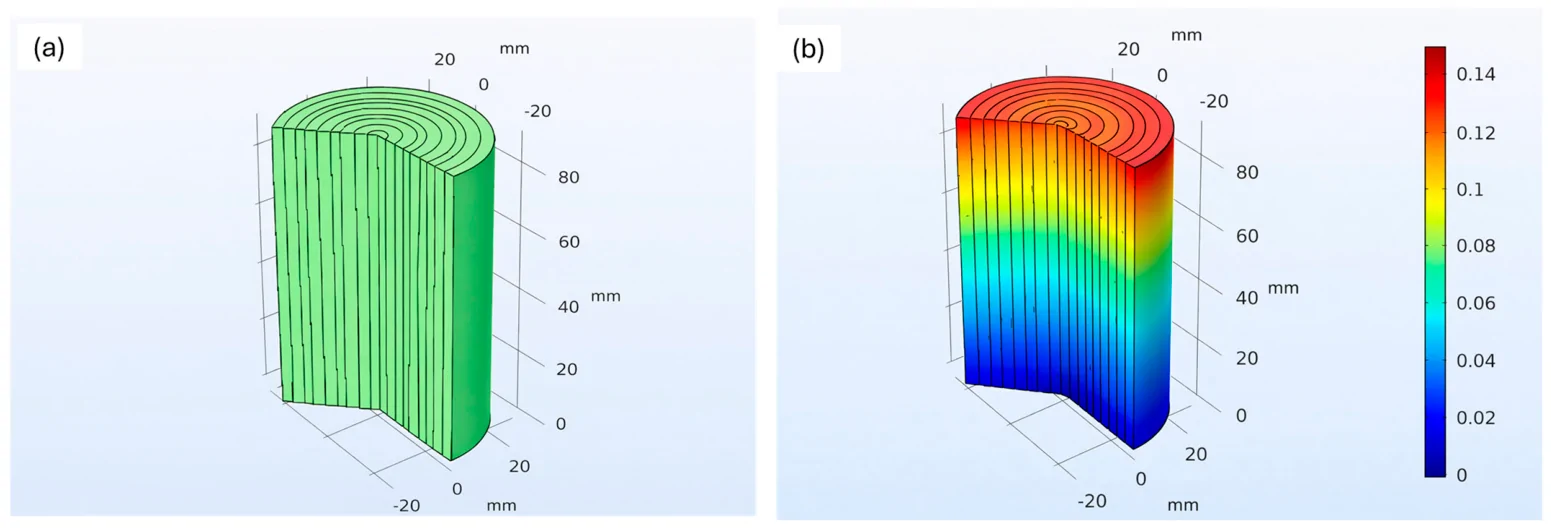
Numerical representation of the CMo cylindrical specimen with assigned material domains: (**a**) undeformed configuration and (**b**) predicted deformed configuration under the applied compression pressure.

The CMo nevertheless remains substantially more deformable than unconfined concrete with a compressive strength of 28 MPa, experiencing greater deformation for a given compressive stress. The numerically predicted deformation pattern of the CMo under compression is shown in Figure 17. Therefore, although incorporating ABS increased the effective compressive stiffness, substantial axial shortening would still need to be considered if the material were used in a structural element such as a column.

### 3.3. Housing Design and Environmental Impact Assessment

Based on the tensile and compressive behavior of the CMo, observed through Models 4 and 6, respectively, structural assessment of the single-story housing system described in Section 2.4.1. and shown in Figure 9 was conducted.

#### 3.3.1. Single-Family Home Design

**Figure 18:**
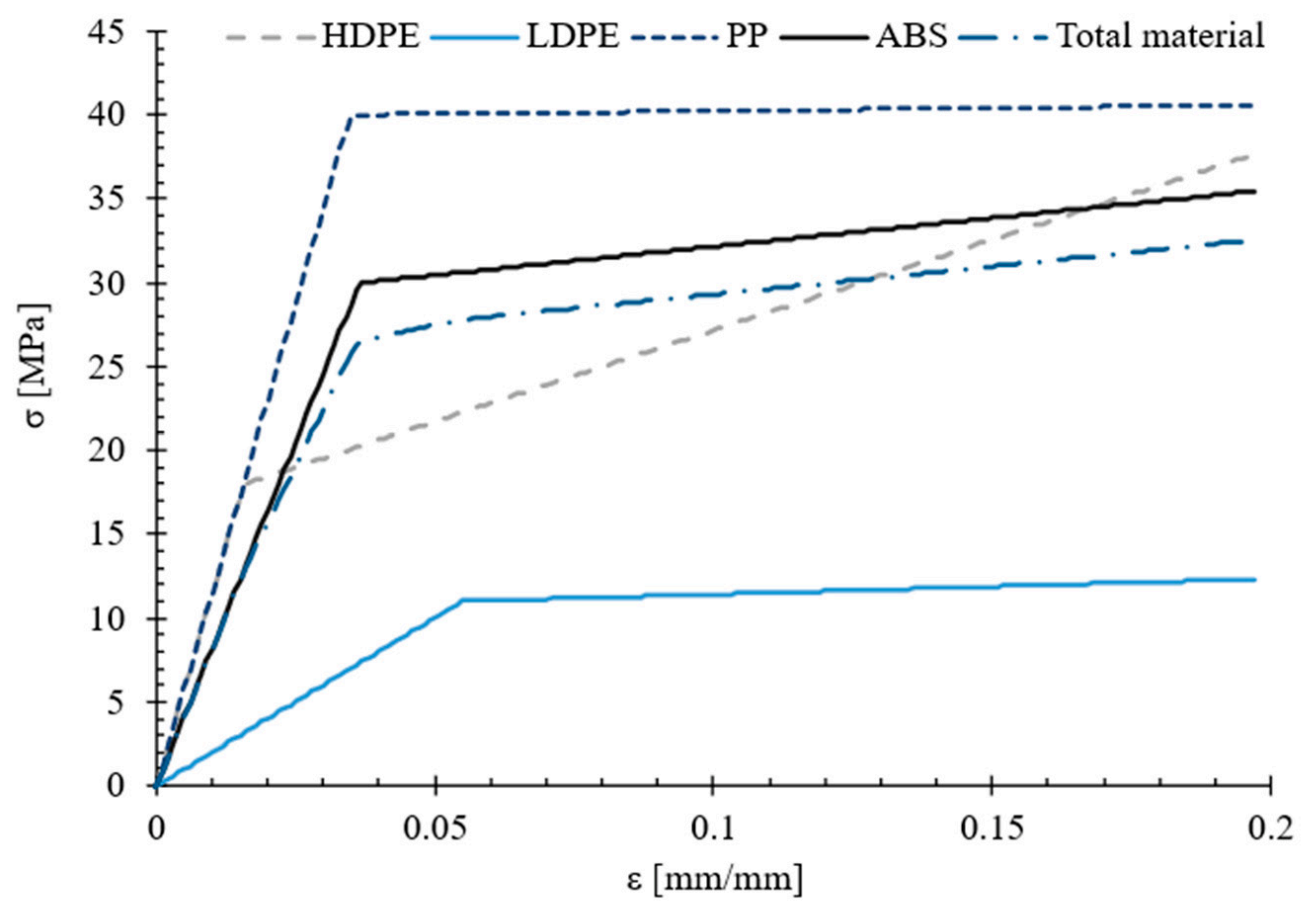
Bilinear compressive stress–strain curves for the constituent polymers and the resulting CMo response (“Total material”) obtained through section analysis.

Definition of compressive properties: The bilinear compressive stress–strain curves constructed from the input parameters in Table 4 for each constituent and for the CMo through the section-analysis procedure described in Section 2.4.1 are presented in Figure 18. For the CMo, defined using assigned section fractions of 13% HDPE, 18% LDPE, 19% PP, and 50% ABS, the calculated curve gives an effective yield stress of 26.30 MPa at a strain of 0.036 mm/mm and reaches an ultimate compressive stress of 32.56 MPa at a strain of 0.197 mm/mm. The reduced post-yield slope represents the idealized strain-hardening response defined by the constituent bilinear models; therefore, these results should not be interpreted as experimental confirmation of the ductility or failure behavior of the CMo.

**Figure 19:**
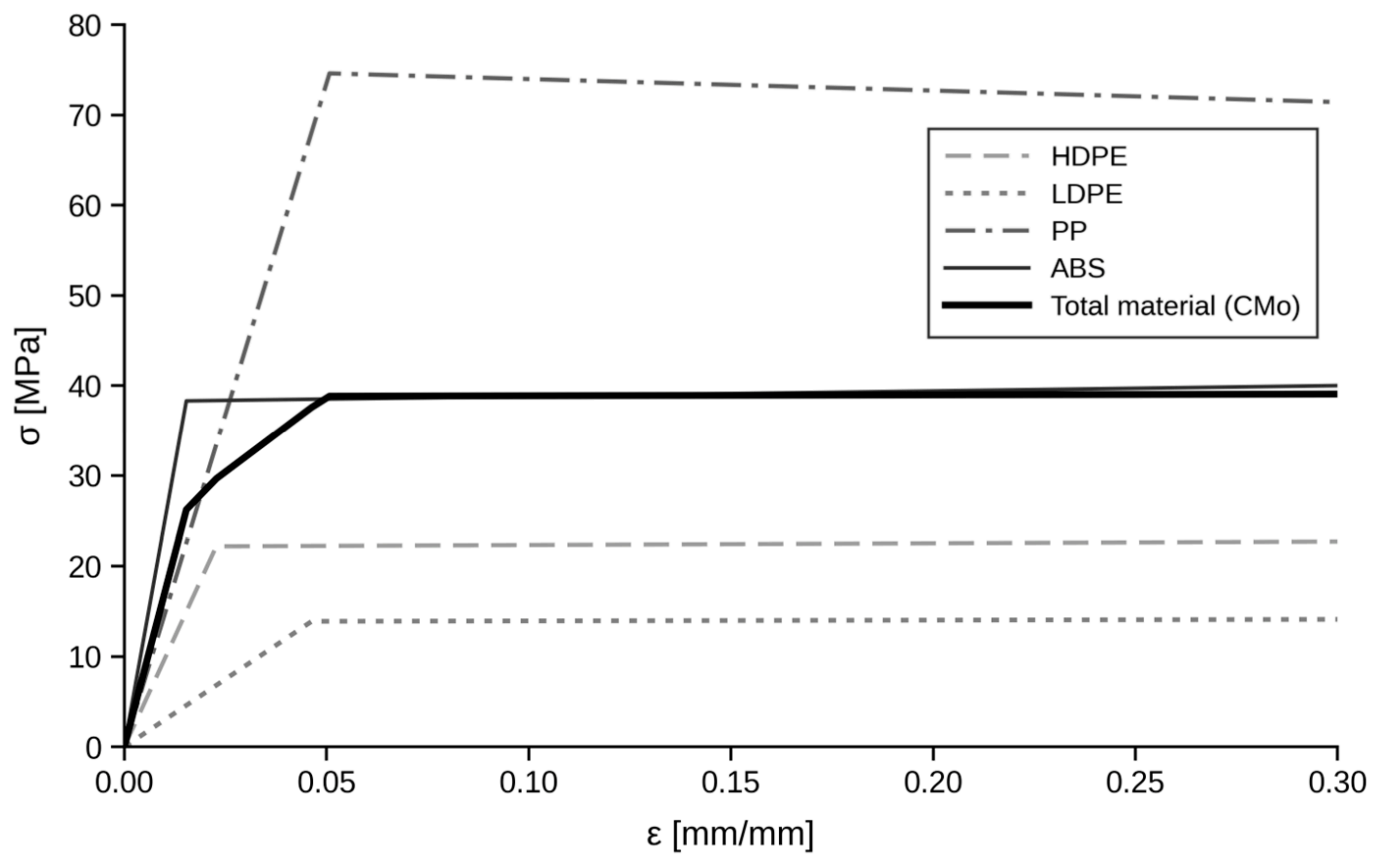
Bilinear tensile stress–strain curves for the constituent polymers and the CMo response (“Total material”) obtained through section analysis.

Definition of tensile properties: The tensile stress–strain curves constructed from the input parameters in Table 4 for each constituent and for the CMo through the section-analysis procedure described in Section 2.4.1 are presented in Figure 19. The calculated CMo curve exhibits an initial linear response with a weighted section-analysis modulus of 1707.7 MPa and first departs from this response at a strain of 0.0154 mm/mm and a stress of 26.21 MPa, corresponding to the onset of yielding in the ABS domain. Between this point and a strain of 0.0507 mm/mm, at which the stress reaches 38.83 MPa, the response has an average secant slope of approximately 356 MPa as the constituent models progressively enter their post-yield branches. Beyond this strain, the curve becomes nearly horizontal, with a slope of about 1.0 MPa, because the post-yield softening assigned to PP offsets the hardening assigned to the remaining constituents; the adopted terminal point is 39.06 MPa at a strain of 0.300 mm/mm, governed by the ultimate strain of the ABS domain. As with the compressive response, these values are analytical estimates and should not be interpreted as experimentally measured yield or failure properties of the CMo.

Structural modeling: Column sections of 0.10 m × 0.10 m were initially considered; however, the preliminary structural analysis led to the adoption of 0.15 m × 0.15 m sections and, for some columns, 0.20 m × 0.20 m sections. For the pre-sizing of the roof purlins, sections of 0.15 m × 0.10 m and 0.15 m × 0.15 m were evaluated. For the maximum purlin spacing of 1.77 m, the *L*/240 criterion described in Section 2.4.1 corresponds to an allowable deflection of 7.37 mm. The calculated minimum section depth was 0.09 m; therefore, the smaller 0.15 m × 0.10 m section satisfied the preliminary deflection criterion. The final member sections used in ETABS^®^ are summarized in Section 2.4.1.

The ETABS^®^ model yielded a fundamental period of 1.13 s and a total modeled mass of 3405.95 kg. For the seismic input parameters summarized in Table 5, the spectral acceleration corresponding to this period was *S_a_* = 0.25*g*, and the calculated seismic shear was 8.35 kN. Under seismic loading, the maximum story-drift ratios were 3.09% in the Y direction and 2.93% in the X direction.

The maximum story-drift ratios obtained from the preliminary analysis exceeded the 1.0% reference limit adopted from NSR-10 [28], as described in Section 2.4.1. This result indicates that the modeled frame did not provide sufficient lateral stiffness under the adopted seismic loading and should therefore not be interpreted as satisfying the reference drift criterion. Future studies should evaluate additional lateral-load-resisting elements, increased member stiffness, or alternative structural configurations, together with experimental characterization of member and connection stiffness, before the seismic performance of the proposed system can be established.

Within the preliminary ETABS^®^ analysis, the model-reported demand-to-capacity ratios for all members remained below 0.80 under the adopted load combinations and provisional capacity-reduction factors described in Section 2.4.1. Accordingly, the selected member sections satisfied the strength checks implemented in the model. These results, together with the comparatively low density of the CMo, indicate its potential for lightweight load-bearing members relative to conventional concrete and structural steel alternatives. Optimized cross-sectional geometries may also improve member stiffness and load-carrying efficiency. However, these results do not establish the overall seismic feasibility of the structural system because the story-drift ratios exceeded the adopted reference limit. Future studies should therefore evaluate braced frames, structural sheathing, or other lateral-load-resisting mechanisms comparable to those commonly used in light-frame timber and cold-formed steel construction, together with full-scale testing of CMo members and connections. The present assessment remains limited to the single-story configuration analyzed.

**Figure 20:**
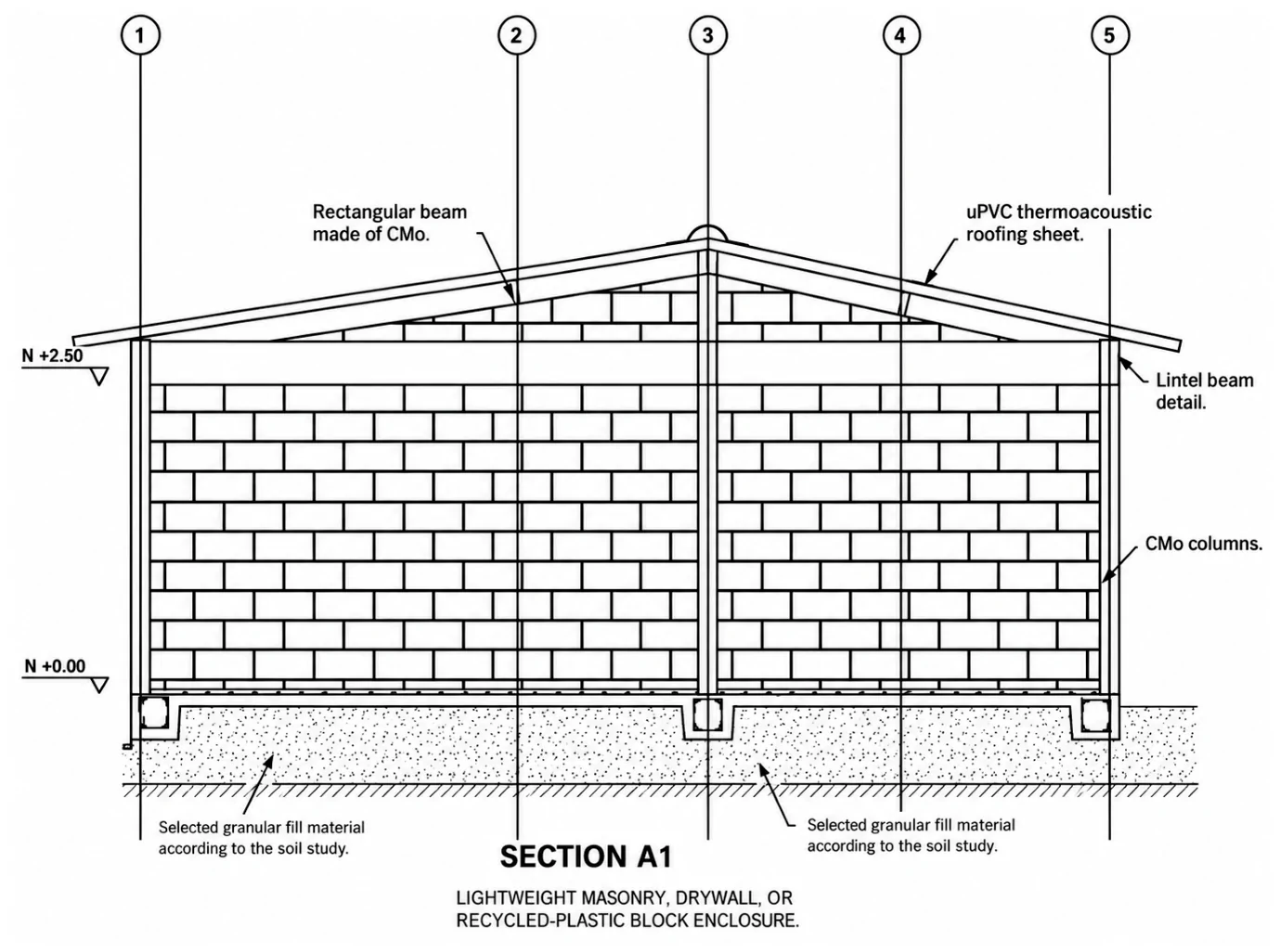
Conceptual cross-section of the proposed single-story housing system showing the CMo structural frame, roof assembly, and nonstructural enclosure.

Proposed design: The conceptual structural system consists of a 2.5 m-high CMo frame with thermoacoustic unplasticized polyvinyl chloride (uPVC) roof sheeting supported by CMo roof-support members with 0.15 m × 0.10 m sections (Figure 20). Lightweight masonry, drywall partitions, or recycled-plastic blocks were considered as enclosure alternatives [29,39]. These enclosure components were treated as nonstructural elements, and their stiffness and strength contributions were not included in the ETABS^®^ model. Consequently, possible interaction between the frame and the enclosure system was outside the scope of the preliminary structural assessment.

**Figure 21:**
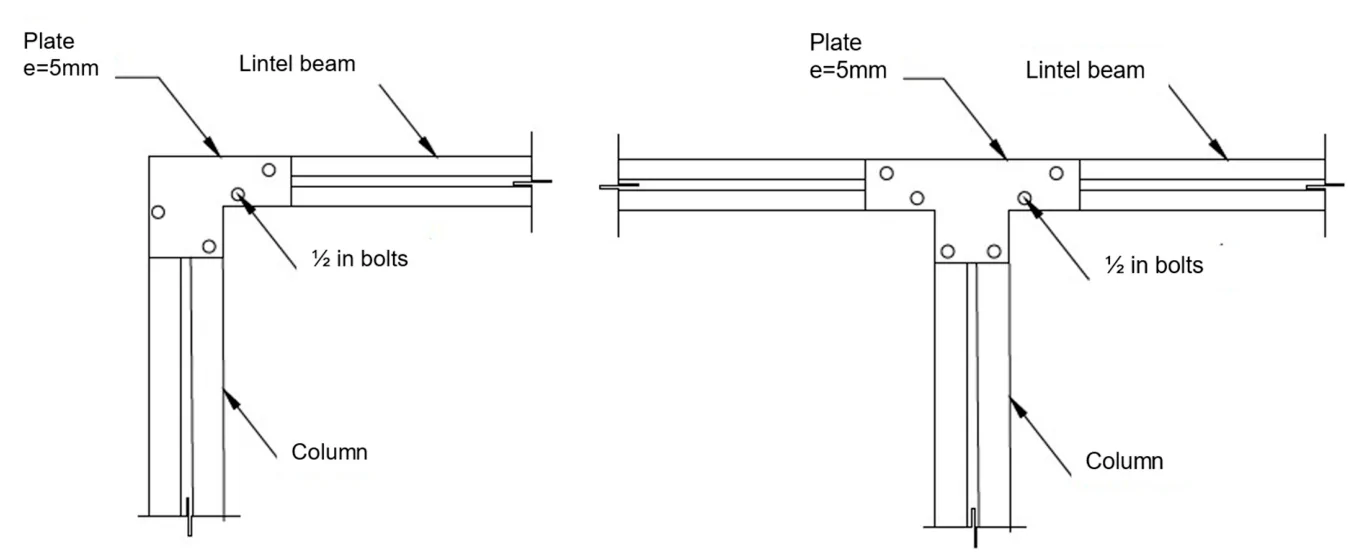
Conceptual beam–column connection details for the proposed CMo structural system.

The proposed CMo columns, lintel beams, and roof-support members were considered as solid sections, with the member dimensions established in Section 2.4.1. For the beam–column connections, 5 mm-thick metal plates and bolts with a nominal diameter of 12.7 mm (1/2 in) are proposed, as illustrated in Figure 21. This connection detail is conceptual; its strength, stiffness, and cyclic performance were not evaluated in the present study and should be addressed through experimental and numerical validation.

#### 3.3.2. Comparison of Estimated CO_2_ Emissions

This section presents findings of the comparative CO_2_ emissions assessment for the proposed single-story housing system. Accordingly, a preliminary estimate of CO_2_-equivalent emissions associated with the constituent materials of the CMo structural elements was obtained following the procedure described in Section 2.4.2. The results were then compared with those for the reference system comprising reinforced-concrete beams and columns and steel roof purlins.

For context, cradle-to-gate CO_2_ emissions of approximately 1.3 and 1.4 kg CO_2_/kg were reported for polyethylene and polypropylene production in Japan, respectively [40]. For recycled LDPE, the adopted emission factor was 0.60 kg CO_2_-eq/kg [35]. For recycled HDPE, a value of 0.63 kg CO_2_-eq/kg was adopted [36]. Regarding recycled ABS, the adopted factor was 0.62 kg CO_2_-eq/kg [37]. Furthermore, the emission factors for ordinary Portland cement, sand, gravel, and water were obtained from Yang et al. [38]. The ITeC database [41] was used as a contextual reference for steel production emissions, whereas the factors adopted for reinforcing steel and structural steel tubing were obtained from worldsteel [33] and ACESCO [34], respectively. These material emission factors are summarized in Table 6. For the separate contribution of in-plant concrete production, Yang et al. report a factor of 0.00768 kg CO_2_/kg of fresh concrete [38].

**Figure 22:**
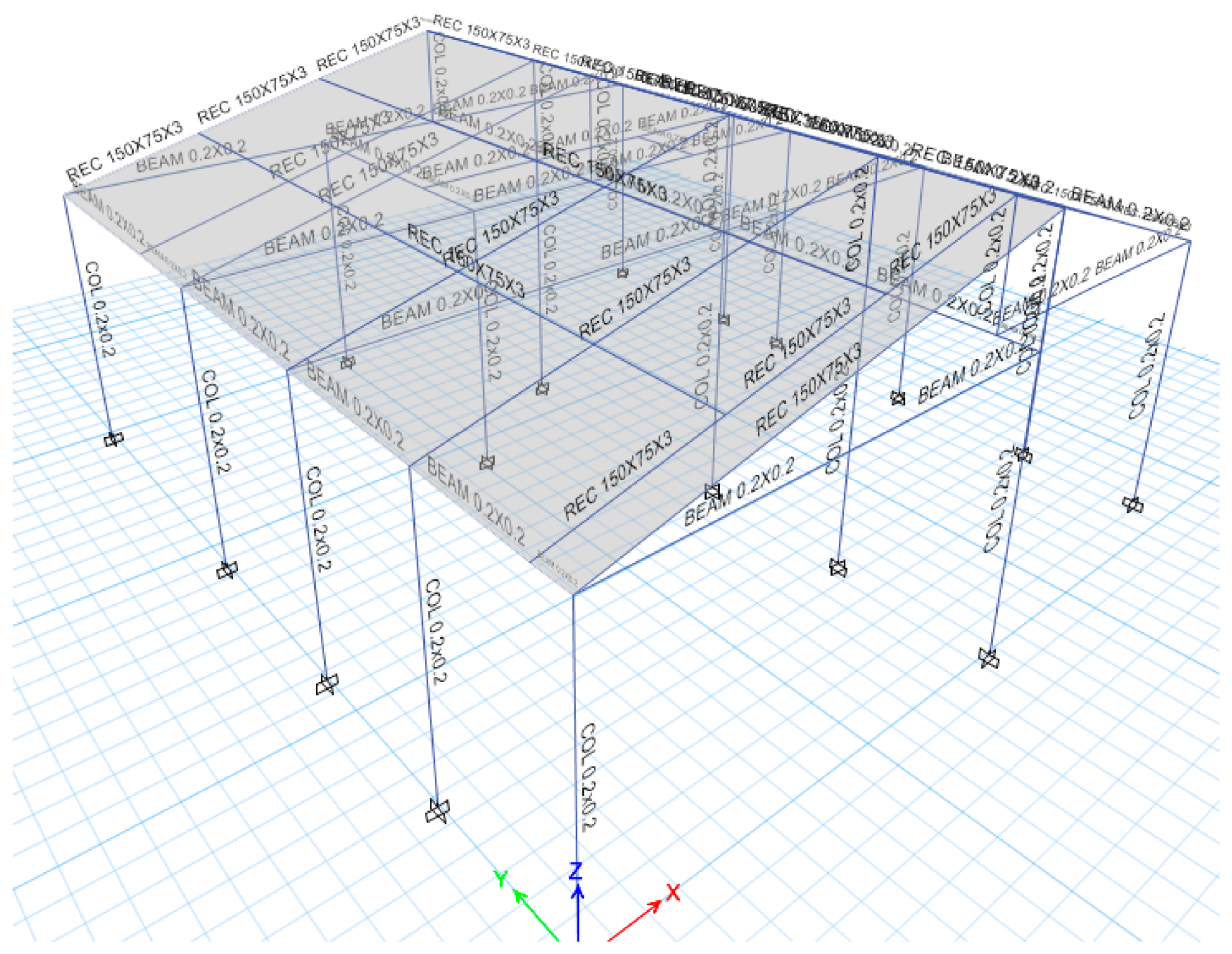
Reference housing structural model comprising a reinforced concrete frame and steel roof purlins.

For comparative purposes, a reinforced concrete frame system was designed as a reference (Figure 22), using concrete with a compressive strength (f’c) of 28 MPa and considering the same architectural requirements and load demands as the proposed CMo housing structure. While the CMo beams were dimensioned with 0.15 m × 0.10 m sections, the reinforced concrete system utilized 0.20 m × 0.20 m beams, reinforced with four longitudinal 9.5 mm (3/8 in) bars and 9.5 mm (3/8 in) stirrups spaced every 0.2 m. For the roof purlins of the reference system, rectangular steel sections measuring 150 mm × 75 mm × 3 mm were considered. Regarding the columns, all reinforced concrete columns have 0.20 m × 0.20 m sections, whereas the CMo system includes five 0.20 m × 0.20 m columns and nineteen 0.15 m × 0.15 m columns.

Initially, the required quantities of concrete and steel (longitudinal and transverse reinforcement, and roof purlins) were estimated as volumes for the reference housing structure. These volumes were then multiplied by the adopted mass densities of 2300 kg/m^3^ for plain concrete and 7800 kg/m^3^ for steel, as listed in Table B.3.2-1 of NSR-10 [28], to calculate the total mass of each material. Table 8 presents the volumes and masses of concrete and steel required to construct the house shown in Figure 22.

The quantities of cement, water, sand, and gravel required to produce concrete depend, among other factors, on the physical properties of the aggregates (e.g., water absorption and specific gravity); for the purposes of this study, data from the literature were used to obtain approximate estimates. A reference mixture for concrete with a target compressive strength of 28 MPa was adopted, consisting of 463.5 kg of cement, 715.82 kg of sand, 913.94 kg of gravel, and 207 kg of water per cubic meter [42]. By multiplying these values by the concrete volume required for the beams and columns of the reference housing structure, reported as 3.91 m^3^ in Table 8, the corresponding material quantities were estimated (Table 9).

Subsequently, the masses of sand, gravel, water, cement, reinforcing steel, and structural steel tubing reported in Table 8 and Table 9 were multiplied by the corresponding emission factors in Table 6 using Equation (6). Reinforcement and roof-purlin tubing were evaluated separately using their respective emission factors. The estimated production-related CO_2_ emissions for the reference structural system were obtained by summing these material contributions and the separate contribution from in-plant concrete production, as expressed in Equation (7).

*Mat_emissions_*_,*i*_ = *m_material_*_, *i*_*EF_i_* (6)

(7) $${\mathrm{Ref}}_{\mathrm{emissions}}=\sum_{i=1}^{6}{\mathrm{Mat}}_{\mathrm{emissions},i}+m_{\mathrm{fresh}}{\mathrm{EF}}_{\mathrm{prod}}$$

where *Mat_emissions_*_,*i*_ denotes the estimated emissions associated with producing material *i* [kg CO_2_-eq], *m_material_*_,*i*_ is its mass [kg], and *EF_i_* is its emission factor [kg CO_2_-eq/kg]. The index *i* represents sand, gravel, water, cement, reinforcing steel, and structural steel tubing. *Ref_emissions_* denotes the total estimated production-related emissions of the reference structural system [kg CO_2_-eq], *m_fresh_* is the total mass of the concrete mixture constituents reported in Table 9 [kg], and *EF_prod_* is the in-plant concrete production emission factor reported by Yang et al. [38] [kg CO_2_/kg], used here as an approximation of CO_2_-equivalent emission factor.

**Figure 23:**
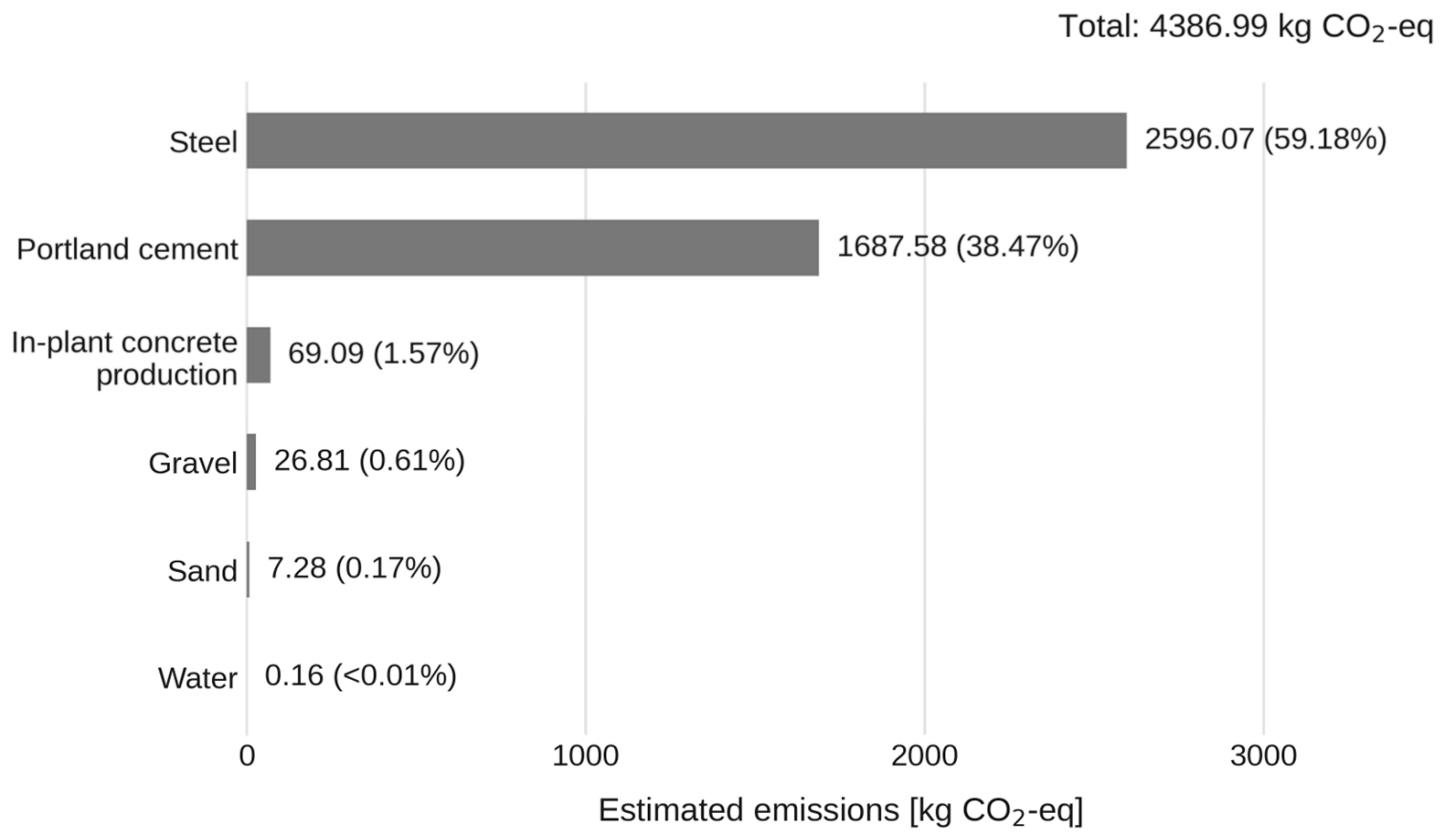
Estimated CO_2_-equivalent emissions by material and in-plant concrete production for the reference structural system under the adopted inventory assumptions. Steel includes reinforcement and roof-purlin tubing. Percentages indicate each contribution’s share of the total.

As shown in Figure 23, the highest estimated CO_2_-equivalent emissions result from the manufacturing of steel and Portland cement, accounting for 59.18% and 38.47% of the total for the reference structural system, respectively. The steel contribution includes reinforcing bars and structural steel tubing. In-plant concrete production contributes 1.57%, while gravel, sand, and water account for 0.61%, 0.17%, and less than 0.01%, respectively. The total estimated emissions are 4386.99 kg CO_2_-eq.

To estimate the CO_2_-equivalent emissions associated with the constituent materials of the CMo structural system, composed of sections with assigned domain fractions of 13% HDPE, 18% LDPE, 19% PP, and 50% ABS, the density of the material was estimated. For this calculation, the assigned domain fractions were treated as volume fractions. Using the densities of HDPE, LDPE, and PP listed in Table 1 and an adopted ABS density of 1070 kg/m^3^, the density of the CMo was calculated using Equation (8).

(8) $$\rho_{\mathrm{CMo}}=\sum_{i=1}^{4}\phi_{i}\rho_{i}$$

where *ρ_CMo_* is the estimated density of the CMo [kg/m^3^]; *ϕ_i_* is the assigned volume fraction of polymer *i*, expressed as a decimal [−]; and *ρ_i_* is its density [kg/m^3^]. The resulting estimated CMo density is 995.10 kg/m^3^.

The volumes of the CMo structural elements were calculated by multiplying their cross-sectional areas by their corresponding cumulative lengths. Table 10 summarizes the volume required for each structural element type and the total volume of CMo.

The estimated CO_2_-equivalent emissions associated with the constituent materials of the CMo structural system were calculated from the mass of each polymer. These masses were obtained by multiplying the total CMo volume reported in Table 10 by the corresponding assigned volume fractions and constituent densities. Equation (6) was then applied using the emission factors in Table 6. The resulting total CMo mass was 3405.98 kg, and the summed estimated emissions were 1800.25 kg CO_2_-eq.

The potential environmental benefit of using the polymers studied in this research in place of reinforced concrete and steel for the main structural elements of housing was evaluated through a preliminary inventory-based comparison. Under the adopted emission-factor assumptions, the estimated emissions were 4386.99 kg CO_2_-eq for the reference structural system and 1800.25 kg CO_2_-eq for the CMo alternative, corresponding to a difference of 2586.74 kg CO_2_-eq (approximately 59%). Given the differing emission metrics and system boundaries of the source datasets, this difference should be interpreted as a screening estimate within the adopted inventory assumptions, rather than as a reduction established through a fully harmonized life-cycle assessment.

## 4. Conclusions

Based on the experimental, computational, and environmental analyses conducted in this study, the main findings are summarized below. These conclusions synthesize the performance of the proposed recycled thermoplastic composite and its potential for structural applications in low-rise housing.

This research evaluated the use of recycled thermoplastics, with approximately 31% of the BM feedstock originating from agro-industrial residues in banana-producing regions. Experimental characterization of the BM provided the basis for inverse identification of its effective composition and subsequent numerical optimization through ABS incorporation to define the CMo.

The BM (26% HDPE, 36% LDPE, 38% PP, expressed as effective mechanically identified fractions) exhibited an experimental tensile elastic modulus of 870.28 MPa. With the incorporation of an assigned ABS domain fraction of 50%, the optimized composite (CMo: 13% HDPE, 18% LDPE, 19% PP, 50% ABS) reached a numerically predicted tensile elastic modulus of 1670.4 MPa, representing a 91.7% increase relative to the numerically identified BM modulus of 871.36 MPa. The predicted effective compressive modulus increased by approximately 6.7%. These results indicate increased elastic stiffness within the adopted numerical models, while the thermal assessment remains limited to temperature distributions under the prescribed boundary conditions.

The preliminary structural assessment indicates the potential of CMo for beams, columns, and roof-support elements in single-story housing systems. Its comparatively low density favors lightweight structural members. The selected sections satisfied the strength checks implemented under the adopted modeling assumptions; however, the story-drift ratios exceeded the 1.0% reference limit, indicating insufficient lateral stiffness of the modeled frame. Although rectangular profiles were studied, future work should evaluate alternative cross-sections (H, I, T) and additional lateral-load-resisting mechanisms, such as bracing or structural sheathing. Full-scale testing of members and connections is required to establish their structural performance.

The preliminary inventory-based comparison yielded approximately 59% lower estimated CO_2_-equivalent emissions for the CMo alternative under the adopted assumptions, indicating a potential environmental benefit. Differences in the emission metrics and system boundaries of the source datasets limit the comparability of the estimates; therefore, this percentage should be interpreted as a screening result.

The combined experimental and numerical approach provides a basis for selecting recycled thermoplastic formulations for further structural investigation. Future research should prioritize the manufacture and experimental characterization of the proposed CMo, including the effects of polymer compatibility and processing conditions, together with the evaluation of creep and long-term durability. A harmonized life-cycle assessment is also needed to refine the environmental comparison. These investigations would complement the structural validation outlined above and support a more complete assessment of the material’s suitability for construction applications.

## Figures and Tables

**Table 1:** Mechanical properties assigned to the polymer constituents in Model 1.

Parameter	LDPE	HDPE	PP
Density [kg/m^3^]	920 ^(a)^	950 ^(a)^	900 ^(a)^
Modulus of elasticity [MPa]	220 ^(a)^	1200 ^(a)^	1400 ^(a)^
Poisson’s ratio [unitless]	0.45 ^(a)^	0.46 ^(a)^	0.40 ^(c)^
Tensile strength [MPa]	20 ^(a)^	28 ^(b)^	25 ^(c)^

^(a)^ Goodfellow [17]; ^(b)^ Asociación Española de Normalización [18]; ^(c)^ Boin et al. [19].

**Table 2:** Thermal conductivity inputs assigned to the polymer constituents in Model 5 [W/m·K].

HDPE	LDPE	PP	ABS
COMSOL material-library value ^(a)^	0.33 ^(b)^	0.22 ^(b)^	0.17 ^(c)^

^(a)^ COMSOL [16]; ^(b)^ Goodfellow [17]; ^(c)^ PROSPECTOR [21].

**Table 3:** Compressive elastic moduli estimated from literature stress–strain curves [MPa].

LDPE	HDPE	PP	ABS
200 ^(a)^	1208 ^(b)^	1150 ^(c)^	821 ^(d)^

^(a)^ Jordan et al. [23]; ^(b)^ Elleuch and Taktak [22]; ^(c)^ Okereke et al. [24]; ^(d)^ Hernandez et al. [25].

**Table 4:** Input parameters used to construct the bilinear stress–strain models for the constituent polymers of the CMo. (**a**) Compression. (**b**) Tension.

Material	Assigned Section Fraction [%]	*E_s_* [MPa]	*f_y_* [MPa]	*ε_y_* [mm/mm]	*ε_u_* [mm/mm]	*f_u_* [MPa]	*E_h_* [MPa]
(**a**) **Compression**							
HDPE	13	1208	18	0.014901	0.20	38.0	108.05
LDPE	18	200	11	0.055000	0.70	17.0	9.30
PP	19	1150	40	0.034783	0.60	42.0	3.54
ABS	50	821	30	0.036541	0.33	40.0	34.08
(**b**) **Tension**							
HDPE	13	976	22.2	0.022746	8.60	38.5	1.90
LDPE	18	300	13.9	0.046333	7.38	20.2	0.86
PP	19	1470	74.6	0.050748	2.65	41.4	−12.77
ABS	50	2495	38.3	0.015351	0.30	40.0	5.97

Compression data sources: HDPE [22]; LDPE [23]; PP [24]; and ABS [25]. Tensile data sources: MatWeb [30].

**Table 5:** Input parameters for structural analysis.

Parameter	Selected Value	NSR—10 Provision [28]
Minimum dead load for residential use with lightweight facades and partitions [kN/m^2^]	2.0	Table B.3.4.3-1
Minimum live load for sloped roofs with a pitch of 15° or less [kN/m^2^]	0.5	Table B.4.2.1-2
Effective peak horizontal acceleration (*A_a_*)	0.1	Figure A.2.3-2
Effective peak horizontal velocity (*A_v_*)	0.1	Figure A.2.3-3
Soil profile type	D	Table A.2.4-1
Short-period site amplification factor (*F_a_*)	1.6	Table A.2.4-3
Intermediate-period site amplification factor (*F_v_*)	2.4	Table A.2.4-4
Usage Group	I	Section A.2.5.1
Importance Coefficient (*I*)	1.0	Table A.2.5-1
Energy dissipation coefficient (*R*)	1.0	-

**Table 7:** Maximum compressive loads and corresponding stresses recorded at test termination for the BM specimens.

Sample	Maximum Applied Load, F [kN]	Loaded Area, A [mm^2^]	Compressive Stress at Test Termination [MPa]
1	76.82	4080.0	18.8
2	76.56	4116.0	18.6
3	76.44	4068.0	18.8

**Table 8:** Quantities of concrete and steel required for the reference housing structural system.

Material	Element	Length, L [m]	Volume, V [m^3^]	Mass, m [kg]
Concrete	Beams of 0.20 m × 0.20 m	63.44	2.54	5836.48
	Columns of 0.20 m × 0.20 m	34.33	1.37	3158.36
	Total concrete		3.91	8994.84
Steel	Rectangular steel roof purlins, 150 mm × 75 mm × 3 mm	69.73	0.09	707.05
	Longitudinal and transverse reinforcement for 0.20 m × 0.20 m beams	269.62	0.03	254.71
	Longitudinal and transverse reinforcement for 0.20 m × 0.20 m columns	145.90	0.03	199.96
	Total steel		0.15	1161.72

**Table 9:** Quantities of materials required for the reference concrete mixture with a target compressive strength of 28 MPa.

Material	Mass for 1 m^3^ of Concrete [kg]	Mass for 3.91 m^3^ of Concrete [kg]
Cement	463.50 ^(1)^	1812.66
Sand	715.82 ^(1)^	2799.43
Gravel	913.94 ^(1)^	3574.24
Water	207.00 ^(1)^	809.54

^(1)^ Culma Pirabán et al., 2017 [42].

**Table 10:** Required volume of CMo for the proposed structural system.

Element	Number of Elements	Cumulative Length [m] *	Volume [m^3^]
Columns of 0.2 m × 0.2 m	5	10.1	0.40
Columns of 0.15 m × 0.15 m	19	32.5	0.73
Lintel beams, purlins, and roof beams of 0.15 m × 0.10 m	64	152.5	2.29
Total CMo	88	195.1	3.42

* Cumulative length is the sum of the individual lengths of all elements included in each row. Volumes are presented to two decimal places; unrounded values were retained for subsequent mass and emission calculations.

## Data Availability

The data supporting the findings of this study are available from the corresponding author upon reasonable request. The data include experimental mechanical test results and numerical simulation outputs generated during the study. Due to the ongoing nature of related research and institutional data management policies, the data are not publicly available.

## Abbreviations

The following abbreviations are used in this manuscript:

ABS	Acrylonitrile Butadiene Styrene
ACI	American Concrete Institute
ASTM	ASTM International
BM	Base Material (composed of PP, HDPE, and LDPE)
CMo	Numerically Optimized Composite Material
CO_2_-eq	Carbon dioxide equivalent
CPE	Chlorinated Polyethylene
DSC	Differential Scanning Calorimetry
FEM	Finite Element Method
FTIR	Fourier-Transform Infrared Spectroscopy
HDPE	High-Density Polyethylene
ITeC	Institut de Tecnologia de la Construcció de Catalunya
LDPE	Low-Density Polyethylene
NSR-10	Colombian earthquake-resistant construction regulations, 2010
OPC	Ordinary Portland Cement
PP	Polypropylene
rABS	Recycled Acrylonitrile Butadiene Styrene
RHDPE	Recycled High-Density Polyethylene
RLDPE	Recycled Low-Density Polyethylene
RPP	Recycled Polypropylene
TGA	Thermogravimetric Analysis
uPVC	Unplasticized Polyvinyl Chloride
